# Level of evidence of enhanced recovery after surgery (ERAS) strategies for elective craniotomy: An updated systematic review

**DOI:** 10.1007/s10143-025-03961-9

**Published:** 2026-01-13

**Authors:** Jacopo Bellomo, Valérie Blom, Vittorio Stumpo, Menno R. Germans, Philipp Bardelli, Luca Regli, Carlo Serra, Victor E. Staartjes, Christian Möhrlen

**Affiliations:** 1 Machine Intelligence in Clinical Neuroscience & Microsurgical Neuroanatomy (MICN) Laboratory, Department of Neurosurgery, Clinical Neuroscience Center, University Hospital Zurich, University of Zurich, Zurich, Switzerland; 2 Institute of Anesthesiology and Perioperative Care Medicine, University Hospital Zurich, Zurich, Switzerland

**Keywords:** Enhanced recovery, Enhanced recovery after surgery, ERAS, Craniotomy, Neurosurgery, Level of evidence

## Abstract

Enhanced Recovery After Surgery (ERAS) protocols offer evidence-based, multimodal strategies to optimize perioperative care and improve outcomes. While extensively studied in other surgical specialties, ERAS implementation in cranial neurosurgery remains underexplored. This study systematically reviews the current evidence on ERAS elements and protocols for elective craniotomy, evaluating their impact on clinical and economic outcomes. An updated systematic review of the literature was conducted using PubMed, Embase, Scopus, and the Cochrane Library. Interventional and observational studies examining pre-, intra-, or postoperative interventions to improve outcomes such as length of stay, complications, or patient satisfaction were included. Two independent reviewers conducted screening, data extraction, and risk-of-bias assessments, with quality grading based on the GRADE framework. Sixty-six studies were included, comprising 39 newly identified and 27 from the prior review. The evidence demonstrated significant heterogeneity in ERAS interventions and study methodologies. Frequently studied elements included preoperative carbohydrate loading and education, intraoperative scalp nerve blocks as well as short-acting and opioid-sparing anesthetic regimens, avoidance of routine wound drains, postoperative early mobilization, clear criteria for same-day or early discharge protocols, avoidance of routine ICU admission, and comprehensive ERAS protocols. Only 33 studies quantitatively reported protocol adherence. According to GRADE assessment, most elements were supported by “low” level of evidence mainly due to heterogeneous methodology, small sample sizes, non-randomized study designs, and limited prospective validation. There has been a significant influx of evidence concerning the application of ERAS in cranial surgery. Our systematic review provides a comprehensive summary of the body of evidence on pre-, intra-, and postoperative interventions as well as established ERAS institutional protocols. While overall evidence quality remains limited by heterogeneity and incomplete adherence reporting, these components may be safe and effective and serve as priority targets for ERAS implementation in elective craniotomy pathways. Future research should focus on multicenter prospective validation, adherence monitoring, and inclusion of underrepresented patient populations to optimize ERAS integration in neurosurgical practice.

**Supplementary Information:**

The online version contains supplementary material available at https://doi.org/10.1007/s10143-025-03961-9.

## Introduction

The Enhanced Recovery After Surgery (ERAS) or fast-track surgery pathway offers a comprehensive, evidence-based perioperative approach designed to enhance clinical outcomes and reduce healthcare costs. A key feature of the ERAS program is its reliance on a multidisciplinary team to develop protocols that integrate multimodal interventions across all phases of surgery, thereby optimizing recovery [1]. 

ERAS interventions, also known as “elements,” are applied throughout the perioperative period. Preoperative measures include patient education (both physical and mental preparation), smoking and alcohol cessation, anemia management, nutritional assessment, reduced nil per os (NPO) time, carbohydrate loading, and the avoidance of benzodiazepines for premedication [2, 3]. Intraoperative strategies emphasize minimally invasive surgery, anesthetic regimens utilizing standardized short-acting sedatives in combination with predominantly short-acting opioids, anesthesia management guided by bispectral index monitoring to prevent prolonged emergence and delirium, optimized anesthesia through local anesthetic techniques, maintenance of normothermia and euvolemia, standardized weight-adjusted antibiotic prophylaxis, the use of pneumatic stockings to prevent deep vein thrombosis (DVT), and fixed prophylaxis against postoperative nausea and vomiting (PONV) [1, 4]. Postoperative care includes extended DVT prophylaxis, multimodal pain and nausea management, avoidance of nasogastric suction and surgical drains, maintaining euvolemia, early mobilization, early removal of catheters and other inserted devices, and the initiation of a high-protein diet [3, 5]. 

The implementation of ERAS protocols has been shown to reduce surgical complications, shorten hospital stays, improve patient satisfaction, and lower healthcare costs, without increasing readmission rates—particularly after the initial learning phase and improved adherence to protocol elements [2, 6–8]. 

ERAS principles are applicable to a wide range of elective surgeries, and the ERAS Society already offers guidelines for several surgical specialties (https://erassociety.org/). However, research on ERAS implementation in cranial neurosurgery remains particularly limited, and specific guidelines for this field have yet to be developed. To address this gap, we conducted a systematic review of ERAS strategies for elective craniotomy in 2021 [6]. Considering recent evidence, it is essential to update this review and present the latest findings, with the aim of providing recommendations on the most studied interventions, based on the quality of the reported evidence.

## Methods

### Aim

We conducted a new systematic review of the literature on both ERAS protocols and ERAS elements in patients undergoing elective craniotomy. The first aim was to update our previous work [6] with the latest published studies. Second, we aimed to provide the readers with additional information regarding the reported adherence as well as the quality of evidence and strength of recommendations associated with the different investigated ERAS interventions.

## Literature search

This work was performed according to the Preferred Reporting Items for Systematic Review and Meta-Analyses (PRISMA) 2020 guideline [9]. The literature search of PubMed/MEDLINE (Ovid), Embase (Ovid), Scopus (Elsevier) and the Cochrane Library databases was conducted on January 4th, 2024. The search was updated on October 9th, 2025. Search strategy exactly replicated that of our previous work, but it was limited to studies published after December 19th, 2019 (Supplemental Methods 1: Search Strategy). Additionally, the reference lists of the final selected articles were screened to identify any pertinent contributions.

## Inclusion and exclusion criteria

Similar to our earlier work, we included all the interventional and observational studies of patients undergoing elective craniotomy for any indication investigating any pre-, intra-, or postoperative intervention targeted at improving at least one of the following outcomes: (1) length of hospital stay, (2) length of ICU stay, (3) postoperative pain, (4) direct and indirect healthcare cost, (5) complication rate, (6) readmission rate, or (7) patient satisfaction. All age groups, sexes and ethnicities were included. Language was restricted to English, Italian, French, Dutch, and German - reflecting the language competencies of the review team. The search was limited to human studies. Case reports, reviews and meta-analyses, congress abstracts, as well as studies comparing two different or more surgical approaches were excluded. The 27 articles reported in the previous review were also included.

## Screening and full text review

Search results were exported to Rayyan online screening system [10] for deduplication and screening. Title, abstract and full text of all the single articles were screened in parallel by two reviewers (J.B. and V.B.). Discrepancies were resolved by discussion and consensus. In case of persistent disagreement, a third reviewer (V.E.S.) was consulted to reach consensus.

## Data extraction

Two reviewers (J.B. and V.B.) simultaneously extracted the data from the included studies with the use of a predefined data collection excel sheet. The following data were extracted: first author, year of publication, study design, sample size, mean patient age and sex distribution, indications for elective craniotomy, number of patients for each treatment arm, elements of the applied ERAS protocol/type of ERAS element, adherence to the ERAS protocol/element (defined as the percentage of actually treated patients compared to the number of patients assigned to that specific treatment), outcome(s) of interest, as well as summary statistics of the effect of ERAS protocol on the outcome(s) of interest (mean ± SD for continuous variable, percentage/absolute risk reduction/relative risk/odds ratio for binary variable).

### Risk-of-Bias assessment and quality of evidence

The risk of bias of each included article was assessed by two reviewers (J.B. and V.B.) using either the Cochrane Risk of Bias In Non-Randomized Studies of Interventions (ROBINS-I)[11] tool or the Revised Cochrane Risk of Bias Tool for Randomized Trials (RoB2) [12]. Quality of evidence and strength of recommendations for the most important interventions was graded by means of the Grading of Recommendations, Assessment, Development, and Evaluations (GRADE) system [13]. Interventions selected for GRADE appraisal represented the most frequently reported ERAS interventions in the included literature and those judged clinically relevant by the authors.

## Results

### Literature search

**Fig. 1:**
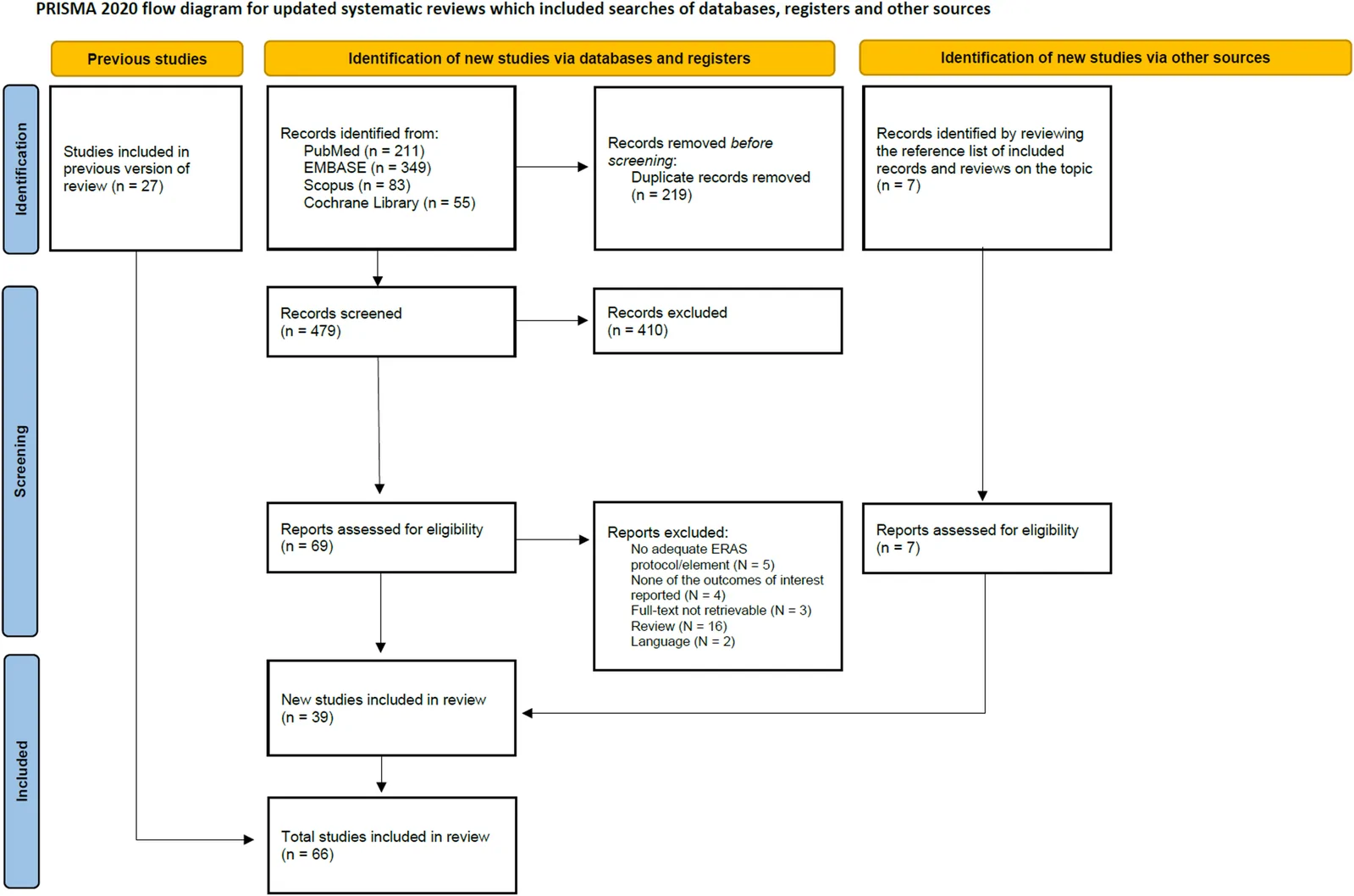
PRISMA 2020 flow diagram the screening and review steps of our updated systematic review of the literature

The literature search (Fig. 1) conducted in PubMed/MEDLINE, Embase, Scopus, and the Cochrane Library yielded 698 articles. An additional seven articles were identified from other sources. After removing duplicates, 479 abstracts were screened, with 410 excluded as irrelevant to the study. Following a full-text review of the remaining 76 articles, 39 were included in the final qualitative analysis (reasons for exclusion during the full-text review are provided in Fig. 1). These were combined with 27 studies from the previous review, bringing the total to 66 studies. Due to the heterogeneity in study methodologies and reported outcomes, quantitative meta-analysis was not feasible. Forty-six studies were interventional, 19 observational. Forty-eight studies reported use of general anesthesia, 6 studies reported the use of awake craniotomy, and 11 studies included both patients undergoing general anesthesia and awake craniotomy.

### Study characteristics

The characteristics of the identified studies, including indications for craniotomy, patient numbers, anesthetic regimens (general vs. awake), demographics, and the adherence to ERAS elements/protocols are detailed in Supplemental Table 1. Our search identified 25 randomized trials, 7 prospective studies, and 34 retrospective studies. Elective craniotomies (supratentorial, *n* = 38; infratentorial, *n* = 4; both supra- and infratentorial, *n* = 16 cor unspecified, *n* = 8) were performed for various pathologies, including intra- and extra-axial tumors, unruptured aneurysms, hydrocephalus, trigeminal neuralgia, hemifacial spasm, cavernous malformations, arteriovenous malformations, and Chiari malformation. Only 33 studies reported data on adherence to the investigated ERAS elements/protocols. The risk of bias for the included studies, assessed using ROBINS-I and RoB2 tools, is also summarized in Supplemental Table 1.

### ERAS elements and outcomes

**Fig. 2:**
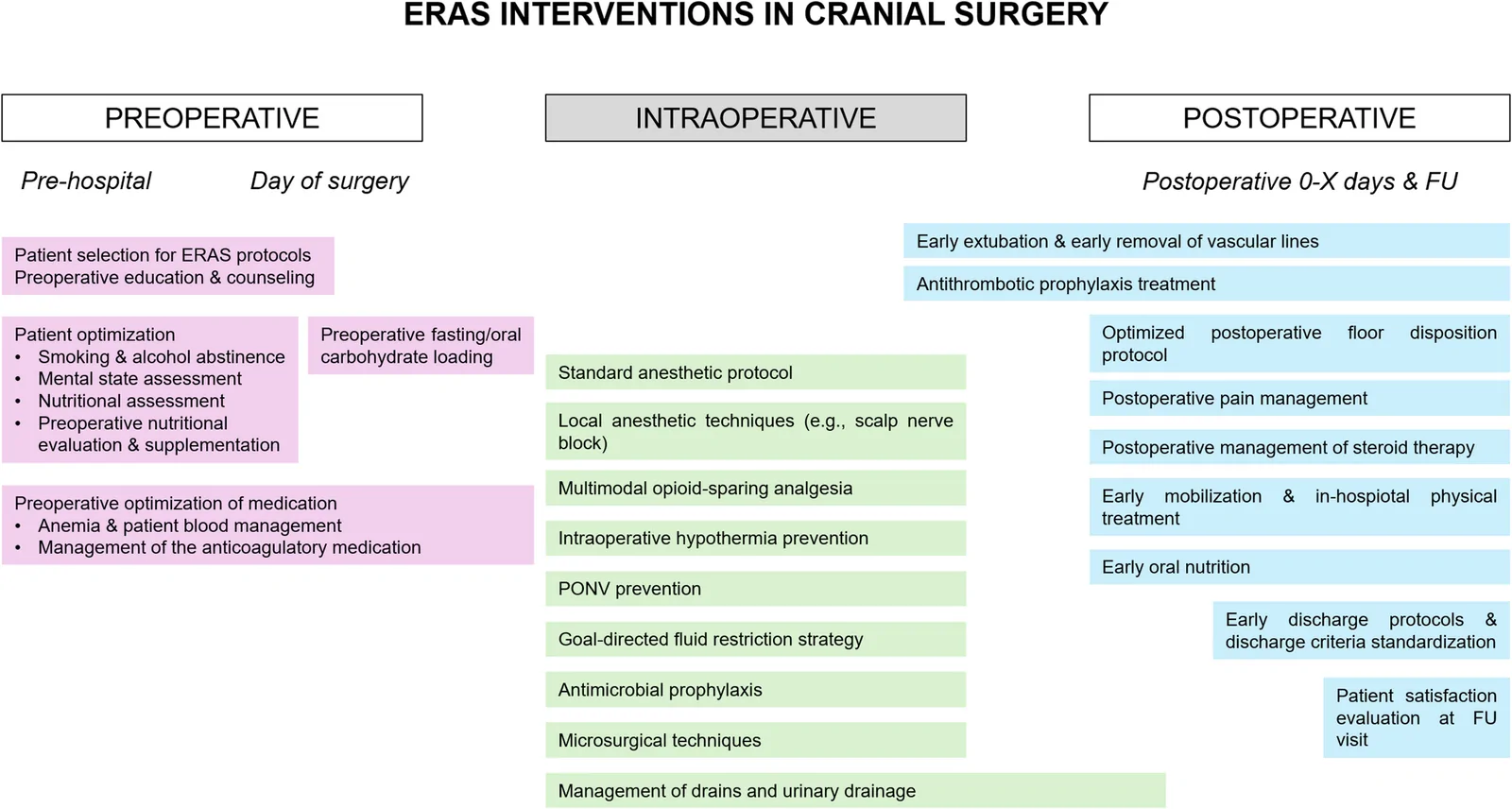
Overview of ERAS interventions in cranial surgery

A total of 22 studies investigating single ERAS elements were identified and are extensively described in Supplemental Tables 2 and presented in Fig. 2. The outcomes associated with these ERAS elements are summarized in Table 1.

**Table 1:** Summary of cranial ERAS elements and investigated outcomes

Element description	Authors & Year	Pathology	Intervention & sample size	LOS (days)	ICU LOS (days)	Postop Complications	Postop Pain	Readmission Rate	Pt Satisfaction	Costs
Preoperative oral carbohydrate loading	Liu et al., 2019[14]	SC/IC for brain tumors, trigeminal neuralgia, cavernoma	Oral carbohydrate loading vs. standard fasting; 58 (int gr) vs. 62 (cont gr)	Median (min, max); 10 (4, 29) vs. 13 (5, 34), ***p*** **= 0.004**	NA	PONV VAS 1–4 84.5% vs. 71.0%; no diff in antiemetic use in VAS > 5; SSI/subcutaneous effusion 1.7% vs. 3.2%; intracranial infection 5.2% vs. 8.1%; Sz 5.2% vs. 3.2%; hemorrhage 3.4% vs. 4.8% respiratory 3.4% vs. 8.1; UTI 0% vs. 1.6%; DVT 0% vs. 3.2%	NA	At postop day 30; 0% vs. 0%	NA	NA
GA regimens	Todd et al., 1993[15]	SC for brain tumors	Propofol (GA ind) + fentanyl-propofol (GA main) vs. thiopental (GA ind) + isoflurane-nitrous oxide (GA main) vs. thiopental (GA ind) + fentanyl-nitrous oxide-isoflurane (GA main); 40 (int gr1) vs. 40 (int gr2) vs. 40 (int gr3)	Mean (SD); 14 (16) vs. 12 (15) vs. 12 (12)	NA	PONV 2.5% vs. 5% vs. 17%, ***p*** **= 0.03**; 2 intracranial hemorrhage vs. 2 Sz, 1 pneumonia, 1 SV tachycardia, 1 PE vs. 1 CE, 1 atrial fibrillation, 1 bone flap infection	NA	NA	NA	Mean (SD); 28.8$ (24.0) vs. 24.0$ (15.4) vs. 25.0 (12.1)
	Gerlach et al., 2003[16]	SC for brain tumors	Remifentanil-propofol vs. sufentanil-propofol for GA ind & main; 18 (int gr1) vs. 18 (int gr2)	NA	NA	PONV 22% vs. 22%; 1 hemiparesis, 1 aphasia vs. 1 hemiparesis, 2 aphasia	Mean (SD) NRS w/in 5 h from surgery; 5.2 (3.6) vs. 5.1 (3.1)	NA	NA	NA
	Djian et al., 2006[17]	SC for brain tumors	Remifentanil-propofol vs. sufentanil-propofol; 29 (int gr1) vs. 31 (int gr2)	NA	NA	PONV 10% vs. 13%	Postop morphine use after surgery; 44.8% vs. 22.6%, ***p*** **= 0.01**	NA	NA	Total anasthesia cost pro pt ◊ 51.9 € vs. 50.2 €
	Bhagat et al., 2008[18]	SC for brain tumors	Propofol vs. fentanyl vs. isoflurane; 47 (int 1) vs. 45 (int 2) vs. 44 (int 3)	NA	Mean (SD); 1.8 (1) vs. 1.5 (1) vs. 1.9 (1)	Sz 2% vs. 2% vs. 5%; hematoma 4% vs. 2% vs. 0%	No postop pain 50% vs. 49% vs. 30%; postop analgesic use 24% vs. 24% vs. 36%	NA	NA	NA
	Uchida et al., 2012[19]	SC for brain tumors	Remifentanil-fentanyl vs. fentanyl alone for analgesia; 936 (int gr) vs. 936 (cont gr)	Median; 17 vs. 19, ***p*** **< 0.001**	NA	In-hospital mortality 1.5% vs. 3.0, ***p*** **= 0.025**	NA	NA	NA	NA
	Bastola et al., 2015[20]	SC for brain tumors	Propofol vs. sevoflurane vs. desflurane for GA main; 25 (int gr1) vs. 25 (int gr2) vs. 25 (int gr3)	NA	NA	PONV 8% vs. 0% vs. 8%; Sz 0% vs. 8% vs. 8%	No post pain 60% vs. 68% vs. 56%; mild 8% vs. 12% vs. 20%; moderate 8% vs. 4% vs. 12%; severe 0% vs. 0% vs. 0%	NA	NA	NA
	Rajan et al., 2016[21]	SC for brain tumors	Dexmedetomidine vs. remifentanil for GA main; 71 (int gr) vs. 71 (cont gr)	NA	NA	NA	Mean (SD); 2.9 (2.6) vs. 5.1 (2.4), ***p*** **< 0.001**	NA	NA	NA
	Negm et al., 2025[22]	SC for various intracranial lesions	Multimodal opioid-sparing analgesia (dexmedetomidine infusion + SNB w/bupivacaine before emergence) vs. conventional opioid-based analgesia; 30 (int gr) vs. 30 (cont gr)	NA	NA	PONV 26.7% vs. 40%, *p* = 0.034	Int gr reported significantly lower VAS scores at 2 h postop (mean ± SD; 2.10 ± 0.69) compared to cont gr (mean ± SD; 3.09 ± 0.68), *p* = 0.039; int gr showed lower pain scores also at 1 h and 4 h. No significant differences were observed at 30 min, 8, 16, or 24 h.	NA	13.3% vs. 36.7% reported “excellent” analgesia (*p* = 0.028)	NA
AC vs. GA	Bojaxhi et al., 2021[23]	SC for brain tumors	AC regimen vs. standard GA regimen; 56 (int gr) vs. 35 (cont gr)	NA	NA	PONV w/in 48 h 75% vs. 88%	Severe pain immediately postop 0% vs. 22.86%, ***p*** **< 0.01** NRS score on POD 0 2.5 vs. 4, ***p*** **< 0.01** Similar pain scores were observed on POD 1 and POD 2	NA	NA	NA
Bilateral SNB before skin incision	Akcil et al., 2017[24]	IC for brain tumors, Arnold-Chiari malformation	SNB w/bupivacaine vs. local anesthesia w/bupivacaine vs. remifentanil alone; 15 (int gr1) vs. 15 (int gr2) vs. 15 (cont gr)	NA	NA	NA	Postop pain lower in SNB gr than cont gr at 10 min, 1 h & 2 h after surgery	NA	NA	NA
	Yang et al., 2019[25]	SC for unruptured aneurysms	SNB w/ropivocaine vs. local anesthesia w/ropivocaine vs. remifentanil alone; 19 (int gr1) vs. 19 (int gr2) vs. 19 (cont gr)	NA	NA	PONV 26% vs. 21% vs. 11%, ***p*** **= 0.017**; fever 6% vs. 11% vs. 6; dizziness 6% vs. 0% vs. 6%	Postop pain lower in SNB gr than in local anesthesia injection and control gr at 2 h, 4 h, 12 h, 24 h & 48 h after surgery	NA	NA	NA
	Yang et al., 2020[26]	NA	SNB w/ropivacaine (different concentrations) vs. sham SNB; 21 (int gr1) vs. 20 (int gr2) vs. 22 (int gr3) vs. 22 (cont gr)	NA	NA	PONV w/in 24 h 19% vs. 25% vs. 31% vs. 31%	For procedures > 4 h, SNB groups exhibit lower postop pain scores than cont group at 2 h, but not at 4 h, 6 h and 24 h; no difference when duration was ≤ 4 h	NA	NA	NA
	Carella et al., 2021[27]	SC for brain tumors, unruptured aneurysms, AVM	SNB w/bupivacaine vs. sham SNB; 30 (int gr) vs. 30 (cont gr)	NA	NA	NA	Postop pain lower in the SNB gr than in the cont gr	NA	NA	NA
	Kadian et al., 2025[28]	SC for various intracranial lesions	SNB w/bupivacaine & ketamine vs. bupivacaine & magnesium sulfate vs. bupivacaine alone; 33(int gr1) vs. 32 (int gr2) vs. 32 (cont gr)	NA	NA	NA	Postop pain as well as the time to first postop analgesic demand was lower in the ketamine group (int gr1) compared to the other groups (*p* < 0.001)	NA	NA	NA
Bilateral SNB before emergence	Hussein et al., 2020	SC for brain tumors	SNB w/bupivacaine-lidocaine-epinephrine vs. fentanyl alone; 15 (int gr) vs. 15 (cont gr)	NA	NA	NA	Postop pain lower in SNB gr than in cont gr at 30 min, 1 h, 2 h, 4 h, 8 h, and 16 h, but not at 24 h	NA	NA	NA
	Rigamonti et al., 2020[29]	SC for brain tumors, unruptured aneurysms, AVM, cavernoma	SNB w/bupivocaine-epinephrine vs. sham SNB; 44 (int gr) vs. 45 (cont gr)	Mean (SD); 4.4 (5.2) vs. 3.6 (3.8)	NA	PONV 29% vs. 25%	Postop pain did not differ between SNB gr and cont gr	NA	PTSS at day 5, mean (SD): 1.8 (2.1) vs. 1.9 (2.3)	NA
	Choi et al., 2023[30]	SC for unruptured aneurysms	SNB w/ropivacaine-epinephrine vs. sham SNB; 27 (int gr) vs. 25 (cont gr)	Median [IQR]; 7 [6, 7] vs. 7 [6, 7]	NA	PONV did not differ between SNB gr and cont gr	Postop pain lower in SNB gr than in cont gr at 3 h, 9 h, and 12 h	NA	NA	NA
Minimally invasive craniotomy	Sarmento et al., 2020[31]	SC for mesial temporal lobe epilepsy	Nummular craniotomy of the temporal bone vs. conventional frontotemporal craniotomy; 43 (int gr) vs. 30 (cont gr)	Mean (SD); 3 (1) vs. 4 (1), ***p*** **< 0.001**	NA	None vs. 4 temporal muscle atrophy, 1 cerebrospinal fluid leakage, and 1 wound infection	NA	NA	NA	NA
Anesthetic injection in the subdural drain	Bala et al., 2022[32]	SC for brain tumors	Injection of ropivacaine in the subdural drain at the end of surgery vs. sham injection; 25 (int gr) vs. 25 (cont gr)	Mean (SD); 11 (4) vs. 11 (7)	NA	No adverse events pertaining to the subdural injection technique observed	Postop pain lower in drain injection gr than in cont gr immediately postop, at 1 h, and at 2 h	NA	NA	NA
Extubation in OR	Cata et al., 2011[33]	IC for brain tumors, vascular pathologies	Obs study; 145	Shorter for pts extubated in OR, ***p*** **= < 0.001**	NA	1 pt reintubated after extubation in OR due to LOC deterioration	NA	NA	NA	NA
Floor disposition	Beauregard and Friedman, 2003[34]	SC/IC for brain tumors, trigeminal neuralgia, Arnold-Chiari malformation	Obs study; 430	Mean; 6.8 vs. 3.8 vs. 3.5, ***p*** **< 0.001**	NA	16% medical, 22% neurosurgical complications (association with floor disposition not investigated)	NA	NA	NA	Mean cost savings of 4’026$ pro pt for floor assignment compared to ICU

AC, awake craniotomy; CE, cerebral edema; cont, control; diff, difference; DVT, deep vein thrombosis; GA, general anesthesia; gr, group; IC, infratentorial craniotomy; ICU, intensive care unit; IMC, intermediate care unit; ind, induction; int, intervention; intraop, intraoperative; LOC, level of consciousness; main, maintenance; ns, no significant; PE, pulmonary embolism; POD, postoperative day; PONV, postoperative nausea and vomitus; postop, postoperative; pt, patient; PTSS, pain treatment satisfaction scale; SC, supratentorial craniotomy; SNB, scalp nerve block; SSI, surgical site infection; SV, supraventricular; sz, seizure; TSS, transsphenoidal surgery; UT, urinary tract

###  Preoperative elements

One study evaluated oral carbohydrate loading as a preoperative intervention and demonstrated a significant reduction in hospital length of stay following elective craniotomy [14]. 

###  Intraoperative elements

Several studies investigated anesthetic strategies, including comparisons between general anesthesia regimens [15–22], the use of awake surgery versus general anesthesia (outside the ERAS protocol context) [23], and adjunctive scalp nerve block (SNB) [22, 24–30]. Most studies on anesthetic regimens were older and heterogeneous in design, limiting the ability to synthesize results or generalize conclusion. In general, the use of short-acting sedatives (e.g., propofol) and short-acting opioids (e.g., fentanyl) has been associated with a lower incidence of PONV [15], while multimodal opioid-sparing analgesia has been shown to reduce both postoperative pain and PONV, although paradoxically with lower reported patient satisfaction [22]. Only one study compared awake surgery versus general anesthesia alone, and not as part of ERAS protocol [23]. Similarly, the investigation into minimally invasive surgical approaches was limited to a single study [31]. Both studies were characterized by relatively small sample size and serious risk of bias. Therefore, less can be said about the influence of these interventions on the selected ERAS outcomes. Seven studies investigated the additional use of SNB to general anesthesia. One study showed that SNB reduced the prevalence of PONV [25], while six studies found that SNB was associated with lower short-term postoperative pain [24–27, 30].

###  Postoperative elements

Two postoperative interventions were evaluated in two studies: the timing of extubation [33] and post-surgery floor disposition [34]. Early extubation in the operating room and the avoidance of routine intensive care unit (ICU) admission were shown to be safe practices with comparable postoperative complication rates when applied to appropriately selected patients.

### ERAS protocols and outcomes

A total of 44 studies investigating ERAS protocols were identified and are described in detail in Supplemental Tables 3 and presented in Fig. 2. The outcomes associated with these ERAS elements are summarized in Table 2.

**Table 2:** Summary of cranial ERAS protocols and investigated outcomes

Protocol description	Authors & Year	Pathology	Sample size	LOS (days)	ICU LOS (days)	Postop Complications	Postop Pain	Readmission Rate	Pt Satisfaction			Costs	
Same-Day Discharge Protocols	Bernstein et al., 2001[35]	SC for brain tumors, unruptured aneurysms	46 (obs study)	NA	NA	1 pt hemiparesis, 1 pt intraop air embolus, 1 pt Sz, 1 pt worsened hemiparesis, 1 pt delayed wound healing	NA	2% ◊ 1 pt HA	NA			NA	
	Boulton & Bernstein, 2008[36]	SC for brain tumors, unruptured aneurysms	262 (obs stury)	NA	NA	2 pts HA, 3 pts Sz, 1 pt hemiparesis, 1 pt air embolus	NA	1.5% ◊ 1 pt HA, 1 pt Sz	NA			NA	
	Grundy et al., 2008[37]	SC for brain tumors, unruptured aneurysms	41 (obs study)	NA	NA	1 pt transient worsening of lower limb function, 1 pt transient worsening of dysphasia	NA	None	NA			NA	
	Purzner et al., 2011[38]	SC for brain tumors, unruptured aneurysms	401 (obs study)	NA	NA	2 pts PONV, 5 pts worsened neurological status, 1 pt air embolus, 1 pt MI, 1 pt Sz, 2 ICH, 1 vasovagal attack	NA	2% ◊ 1 pt worsened neurological deficit, 2 pts ICH, 1 pt PONV, 2 pts Sz, 1 pt HA	NA			$2’313’960 saved for 401 pts	
	Goettel et al., 2014[39]	SC for brain tumors, unruptured aneurysms	25 (obs study)	NA	NA	1 pt vasospasm; 4 pts transferred to PACU w/decreased LOC (*n* = 1), bradycardia (*n* = 1), fever (*n* = 1), severe PONV (*n* = 1); 1 pt to NCCU w/decreased LOC; 1 pt to ICU w/Sz; 2 pts to the ward w/severe postop pain (*n* = 1), transient motor weakness (*n* = 1)	NA	4% ◊ 1 pt vasospasm	NA			NA	
	Au et al., 2016[40]	SC for brain tumors, unruptured aneurysms	44 (obs study)	NA	NA	1 pt Sz, 1 pt incision oozing, 1 pt cognitive impairment, 1 pt new hemiparesis, 1 pt worsened hemiparesis, 1 pt dysphasia	NA	2% ◊ 1 pt new aphasia	NA			NA	
	Venkatragh-avan et al., 2016[41]	SC for brain tumors, unruptured aneurysms	198 (obs study)	NA	NA	9 pts new or worsened neurological deficits, 4 pts Sz, 4 pts HA w/PONV, 6 other reasons	Mean (SD) postop opioid consumption in PACU (mg, expressed as morphine equivalents); 16 (17)	3% ◊ 1 pt Sz, 1 pt confusion, 1 pt new hemiparesis, 1 pt HA, 1 pt bleeding wound	NA			NA	
	Nassiri et al., 2019[42]	SC for brain tumors, unruptured aneurysms	29 (obs study)	NA	NA	2 pts Sz, 2 pts VTE, 4 pts SSI	NA	17% ◊ 4 pts SSI, 1 pt Sz	NA			Mean total hospital costs per pt ◊ $5’242	
	Nijs et al., 2023[43]	SC for brain tumors, unruptured aneurysms	630 (obs study)	NA	NA	28 pts new neurological deficit; 8 pts Sz; 12 pts PONV; 5 pts HA	NA	1.4%	NA			NA	
	Levy et al., 2023[44]	SC for brain tumors	31 (int gr) vs. 59 (cont gr)	NA	NA	1pt transient neurological deficit and 1 pt postop ICH in the cont gr	NA	10% vs. 14%	NA			NA	
	Peláez-Sanchez et al., 2023[45]	SC for brain tumors	18 (int gr) vs. 59 (cont gr)	NA	NA	1 pt new neurological deficit, 2 pts ICH vs. 2 pts new neurological deficit, 5 pts ICH, 2 pts Sz	NA	0% vs. 2%	NA			NA	
	Peláez-Sanchez et al., 2025[46]	SC for brain tumors	18 (int gr) vs. 38 (cont gr)	NA	NA	NA	NA	NA	NA			Mean (SD) total cost per pt ◊ 4468 (889) € vs. 7327 (1430) €, *p* < 0.001	
	Gurses et al., 2024[47]	SC for brain tumors	202 (obs study)	NA	NA	1 pt hypoxia, 1 pt episode of left-sided incoordination, 1 pt PONV, 1 pt ICH, 1 pt cranial nerve palsy, 1 pt acute kidney injury, 1 pt SSI, 1 pt seizure	NA	6.4% ◊ 1 pt septic shock, 1 pt hyperglycemia, 1 pt stroke-like migraine after radiation therapy, 1 pt dehydration, 1 pt pain, 1 pt diverticulitis, 1 pt hyponatremia, 1 pt altered mental status, 1 pt fever, 1 pt nausea&vomiting, 1 pt hydrocephalus, 1 pt aphasia, 1 pt visual disturbancies	NA			NA	
Early Discharge Protocols	Sughrue et al., 2015[48]	SC/IC for brain tumors	213 (obs study)	60% 1 day, 40% 2 days	NA	3 pts CSF disorder, 2 pts infection, 2 pts steroid withdrawal, 1 pt Sz, 1 pt pneumencephalon	NA	4% ◊ 3 pts CSF disorder, 2 pts infection, 2 pts steroid withdrawal, 1 pt Sz, 1 pt pneumenceph-alon	NA			NA	
	Ma et al., 2016[49]	SC for brain tumors	75 (obs study)	Median (min, max); 1 (1, 49)	NA	7 pts wound infection, 2 pts wound dehiscence	NA	16%	NA			Cost savings per pt of £2’928	
	Neville et al., 2020[50]	SC/IC for brain tumors	34 (int gr) vs. 32 (cont gr)	Median [IQR]; 3 [2, 5] vs. 5 [4, 8], ***p*** **= 0.001**	Median [IQR]; 1.1 [1.0, 2.1] vs. 1.2 [0.9, 2.8]	1 pt periop stroke, 3 pts severe sepsis, 1 pt UT, 1 pt DVT, 3 pts Sz vs. 1 pt periop stroke, 1 pt PE, 5 pts severe sepsis, 2 pts meningitis, 2 pts status epilepticus, 1 pt UT, 2 pts DVT, 1 pt SSI, 2 pts wound dehiscence, 4 pts CSF fistula, 4 pts Sz 30-day mortality ◊ 2 pts vs. 1 pt	NA	9.4% vs. 18.8%, *p* ns ◊	NA			Median [IQR] hospital costs per pt ◊ $2’135 [1’472, 3’800] vs. $2’765 [2’185, 4’333], ***p*** **= 0.043**	
Avoidance of Routine ICU Admission Protocols	Ter Laan et al., 2020[51]	SC for brain tumors	109 (int gr) vs. 107 (cont gr)	Mean; 5.17 vs. 6.8	Mean; 0.23 vs. 0.47, ***p*** **< 0.05**	Serious complications ◊ 3.7% vs. 5.6%, ***p*** **< 0.001**	NA	NA	Qualitative analysis on 12 pts ◊ pts satisfied			Mean total hospital costs per pt ◊ €11’654 vs. €13’607	
	Hoffman et al., 2023[52]	SC for brain tumors	202 (int gr) vs. 193 (cont gr)	Median [IQR] total LOS; 1.7 [1.0, 2.1] vs. 2.0 [1.6, 3.8], ***p*** **< 0.001**	NA	1 pt DVT vs. 7 pts DVT or UT	NA	19% vs. 18%	NA			Median [IQR] total hospital costs per pt ◊ $138’044 [117’350, 163’095] vs. $152’109 [129’933, 185’628], ***p*** **< 0.001**	
	Perez-Vega et al., 2023[53]	Brain tumors	57 (obs study)	Mean (SD) postop LOS; 1.45 (0.81)	NA	No postop complications were reported	NA	NA	NA			Mean estimated cost savings per pt ◊ $2’600	
	Wagner et al., 2025[54]	SC & IC for brain tumors and vascular lesions	5638 (obs study)	NA	NA	Surgical complications ◊ 964 pts (17.1%): 694 pts new neurological deficit, 361 pts rebleeding, 239 pts thromboembolism; revision surgery ◊ 261 pts (4.6%); PACU-ICU transfer ◊ 106 pts (1.9%): 61 pts prolonged decreased level of consciousness, 28 pats agitation, 17 pts severe neurological disability	NA	NA	NA			NA	
Awake Craniotomy Protocol	Chen et al., 2021[55]	Brain tumors, trigeminal neuralgia	20 (obs study)	Median postop LOS; 9.5	Median; 1	1 pt Sz, 3 pts severe postop pain, 2 pts PONV	NA	0%	NA			NA	
	Azghadi et al., 2025[56]	NA	23 (int gr) vs. 38 (cont gr)	Median [IQR] total LOS; 6.0 [3.0, 8.5] vs. 5.0 [3.0, 8.0]	NA	3 pts vs. 6 pts PONV	No differences between the control and mERAS groups regarding postop pain consumption	NA	NA			NA	
Process Improvement Protocols	McLaughlin et al., 2014[57]	IC for trigeminal neuralgia, hemifacial spasm	25 (int gr1) vs. 7 (int gr2) vs. 12 (cont gr)	Mean; 3.27 vs. 2.12 vs. 3.16, ***p*** **< 0.001**	No. of pts w/o ICU ◊ 2/25 pts vs. 3/7 pts vs. 0/12 pt, ***p*** **= 0.036**	1 transient facial weakness & loss of hearing & possible rhinorrhea, 1 ARDS, 1 angina, 1 chemical meningitis vs. none vs. 1 pt Bell’s palsy, 2 pts CSF leaks	NA	0% vs. 0% vs. 2%	NA			Mean total hospital costs per pt ◊ $23’161 vs. $18’480 vs. $24’771	
	McLaughlin et al., 2014[58]	IC for trigeminal neuralgia, hemifacial spasm	29 (int gr) vs. 20 (cont gr)	Mean; 2.59 vs. 3.05, ***p*** **= 0.041**	Mean; 1.4 vs. 1.1	1 pt decresed hearing, 1 pt transient facial weakness, 1 pt CSF leak, 1 pt chemical meningitis vs. 1 pt decreased hearing, 1 pt transient facial weakness, 3 pts CSF leak	NA	3.4% vs. 15%	NA			NA	
Early Mobilization Protocols	Chen et al., 2025[59]	SC/IC for glioma	59 (int gr) vs. 56 (cont gr)	Mean (SD); 12.41 (2.65) vs. 14.43 (3.14), *p* < 0.001	NA	NA	NA	NA	NA			Mean (SD); 32,883 (2635) vs. 39,824 (11698), *p* < 0.001	
	Zarrin et al., 2025[60]	Craniotomy for brain tumors	1059 (obs)	Mean; 2.9	NA	NA	NA	30-day readmission rate ◊ 6%	NA			Mean total hospital costs; $104’050	
Comprehensive ERAS Protocols	Wang et al., 2018[61]	SC/IC for brain tumors, trigeminal neuralgia, hemifacial spasm	70 (int gr) vs. 70 (cont gr)	Median [IQR]; 10 [7, 14] vs. 13 [10, 18], ***p*** **= 0.004**	NA	SSI/subcutaneous effusion 3 pts vs. 2 pts; intracranial infection 4 pts vs. 6 pts; Sz 3 pts vs. 2 pts; hemorrhage 2 pts vs. 3 pts; respiratory 2 pts vs. 5 pts; CVD 1 pt vs. 0 pt; UT 0 pt vs. 1 pt; DVT 0 pt vs. 2 pts	POD 1 VAS score 1–3 ◊ 55 pts vs. 23 pts; 4–6 ◊ 13 pts vs. 42 pts; 7–9 ◊ 2 pts vs. 5 pts (***p*** **< 0.001**)	0% vs. 0%	Mea (SD); 92.2 (4.3) vs. 86.8 (7.4), ***p*** **< 0.001**			Median [IQR] total hospital costs per pt ◊ $7’591 [5’119, 13’121] vs. $9’224 [6’450, 19’956]	
	Liu et al., 2019[62]	SC/IC for brain tumors, trigeminal neuralgia, hemifacial spasm	70 (int gr) vs. 70 (cont gr)	NA	NA	NA	NA	NA	Overall satisfaction ◊ 92.2 vs. 86.8, ***p*** **< 0.001**; information ◊ 17.4 vs. 16.5, ***p*** **= 0.039**; medical care ◊ 18.9 vs. 18.3, ***p*** **= 0.043**; nursing care ◊ 19.2 vs. 18.6, ***p*** **= 0.032**; enhanced recovery ◊ 18.5 vs. 15.7, ***p*** **< 0.001**; comfort & others ◊ 18.2 vs. 17.9			NA	
	Liu et al., 2020[63]	SC/IC for brain tumors	36 (int gr) vs. 29 (cont gr)	NA	NA	2 pts intracranial infection, 3 pts Sz, 2 pts hemorrhage, 1 pt respiratory, 1 pt CVD vs. 1 pt SSI/subcutaneous effusion, 2 pts intracranial infection, 2 pts Sz, 2 pts hemorrhage, 1 pt stroke, 3 pts respiratory, 1 pt UT, 2 pts DVT	NA	NA	EORTC QLQ-C30 at discharge; mean (SD) scores for different domains ◊ role functioning 83.9 (21.4) vs. 71.2 (20.0), ***p*** **= 0.017**; constipation 15.4 (20.3) vs. 26.7 (15.2), ***p*** **= 0.016**; no differences in remaining domains EORTC QLQ-C30 at 3-mo FU; mean (SD) scores for different domains ◊ global health status 74.8 (17.4) vs. 64.0 (22.0), ***p*** **= 0.035**; no differences in remaining domains EORTC QLQ-C30 at 6-mo FU; mean (SD) scores for different domains ◊ physical functioning 85.8 (21.9) vs. 74.2 (22.0), ***p*** **= 0.038**; no differences in remaining domains EORTC QLQ-BN20 at discharge; mean (SD) scores for different domains ◊ motor dysfunction 33.2 (25.2) vs. 45.2 (21.4), ***p*** **= 0.047**; no differences in remaining domains EORTC QLQ-BN20 at 3-mo FU; mean (SD) scores for different domains ◊ motor dysfunction 30.2 (30.4) vs. 45.9 (20.3), ***p*** **= 0.020**; drowsiness 20.1 (21.6) vs. 30.5 (18.9), ***p*** **= 0.046**; weakness of legs 27.3 (20.0) vs. 38.7 (15.3), ***p*** **= 0.014** EORTC QLQ-BN20 at 6-mo FU; mean (SD) scores for different domains ◊ motor dysfunction 30.7 (22.1) vs. 43.9 (21.9), ***p*** **= 0.019**; drowsiness 20.5 (17.0) vs. 32.7 (17.5), ***p*** **= 0.006**			NA	
	Qu et al., 2020[64]	SC/IC for brain tumors	64 (int gr) vs. 65 (cont gr)	Median [IQR] total LOS; 10 [8, 12] vs. 13 [11, 17], ***p*** **= 0.004**	NA	9 pts fever, 3 pts Sz, 4 pts hyperglycemia, 10 pts PONV vs. 14 pts fever, 2 Sz, 1 pt raised ICP, 1 pt mental status changes, 3 pts hyperglycemia, 18 pts PONV	Mean (min, max) NRS on POD 1 ◊ 3.1 (1, 8) vs. 4.4 (1, 9), ***p*** **= 0.01**; POD 2 ◊ 2.9 (0, 6) vs. 4.3 (0, 8), ***p*** **= 0.002**; POD 3 ◊ 2.3 (0, 5) vs. 4.0 (0, 6), ***p*** **< 0.01**; POD 4 ◊ 2.3 (0, 4) vs. 2.8 (0, 6)	0% vs. 0%	NA			Median [IQR] total hospital costs per pt ◊ $7’247 [4’652, 16’445] vs. $8’911 [5’525, 19’521], ***p*** **< 0.001**	
	Wang et al., 2022[65]	SC/IC for brain tumors	80 (int gr) vs. 71 (cont gr)	Median [IQR] total LOS; 7 [6, 12] vs. 9 [8, 14], ***p*** **< 0.001**	NA	1 pt SSI/subcutaneous effusion, 2 pts intracranial infection, 1 pt Sz, 1 intracranial hypertension, 1 pt respiratory, 1 pt UT, 6 PONV vs. 2 pts SSI/subcutaneous effusion, 3 pts intracranial infection, 3 pts Sz, 1 pt hemorrhage, 2 pts intracranial hypertension, 1 pt CSF leakage, 4 pts respiratory, 1 pt UT, 13 pts PONV No mortality was observed in both groups	NA	0% vs. 0%	NA			Median [IQR] total hospital costs per pt ◊ $10’578 [9’641, 12’330] vs. $11’343 [10’299, 12’625]	
	Wang et al., 2022[66]	SC/IC for brain tumors, trigeminal neuralgia, hemifacial spasm	76 (int gr) vs. 75 (cont gr)	Median [IQR] postop LOS; 3 [2, 4] vs. 4 [4, 6], ***p*** **< 0.001**	NA	PONV ◊ 7 pts vs. 21 pts, ***p*** **= 0.003** Other non-surgical ◊ 1 pt postop delirium, 1 pt respiratory vs. 3 pts postop delirium, 6 pts respiratory	Mean (SD) postop NRS at 24 h ◊ 1.5 (1.2) vs. 2.6 (1.3), ***p*** **< 0.001**; 48 h ◊ 0.7 (0.8) vs. 1.7 (0.9), ***p*** **< 0.001**; 72 h ◊ 0.4 (0.7) vs. 1.7 (1.5), ***p*** **< 0.001**	0% vs. 0%	NA			Median [IQR] total hospital costs per pt ◊ $5’880 [5’603, 7’024] vs. $6’266 [6031, 8961], ***p*** **< 0.001**	
	Yakar et al., 2023[67]	SC for unruptured aneurysms	38 institutions (obs study)	Mean total LOS; <4 ◊ 3 sites; 4–10 ◊ 28 sites; >10 ◊ 7 sites	NA	Complication rate ≤ 5% ◊ 24 sites; >5% ◊ 6 sites	NRS moderate to severe ≤ 5% of pts ◊ 11 sites; >5% of pts ◊ 27 sites	≤ 5% ◊ 32 sites; >5% ◊ 6 sites	NA			NA	
	Wu et al., 2021[68]	SC for brain tumors	76 (int gr) vs. 75 (cont gr)	Mean (SD) postop LOS; 12.5 (3.5) vs. 13.4 (3.3)	NA	2 pts intracranial infection, 1 pt UT, 1 pt electrolyte disorders, 2 pts pleural effusion vs. 3 pts intracranial infection, 5 pts lung infection, 2 pts UT, 3 pts electrolyte disorders, 1 pt DVT, 2 pts pleural effusion, 2 pts hypoglycemia No mortality was observed in the two groups	NA	NA	NA			Mean (SD) total hpspital costs per pt ◊ $7’065.26 (1’961.41) vs. $8’003.23 (1970.47), ***p*** **= 0.004**	
	Elayat et al., 2021[69]	SC for brain tumors	35 (int gr) vs. 35 (cont gr)	Mean (SD) total LOS; 11.5 (9.0) vs. 12.1 (8.8)	No of pts staying at ICU > 48 h ◊ 15 vs. 27, ***p*** **= 0.003**	No complications in the ERAS group; 1 pt brain infarction, 2 pts CE, 1 pt transient renal failure, 1 pt septic shock in the cont gr	No. of pts with at least 1 episode VAS > 4 w/in 48 h after surgery ◊ 7 vs. 16, ***p*** **= 0.02**	NA	NA			NA	
	Feng et al., 2021[70]	SC/IC for brain tumors	50 (int gr) vs. 50 (cont gr)	Median total LOS; 13.0 vs. 15.5, ***p*** **< 0.001**	NA	1 pt intracranial infection, 2 pts neurological dysfunction, 1 pt Sz, 2 pts pneumonia, 1 pt DVT vs. 2 pts intracranial infection, 2 pts neurological dysfunction, 1 pt Sz, 2 pneumonia, 2 DVT	Postop mild pain (NRS 0–3) ◊ 38 pts vs. 24 pts, ***p*** **= 0.004**; moderate pain (NRS 4–6) ◊ 9 pts vs. 19 pts, ***p*** **= 0.026**; severe pain (NRS 7–10) ◊ 3 pts vs. 7 pts	NA	NA			Mean (SD) total hospital costs per pt ◊ $8’038 (1’063) vs. $8’960 (1’694), ***p*** **< 0.001**	
	Abhinav et al., 2023[71]	SC/IC for brain tumors	128 (int gr) vs. 130 (cont gr)	Mean total LOS; 10 vs. 13, ***p*** **= 0.04**	NA	NA	Mean (SD) postop NRS on POD1 ◊ 3.13 (0.21) vs. 4.45 (0.95), ***p*** **= 0.011**; POD3 ◊ 2.33 (0.52) vs. 4.04 (0.15), ***p*** **< 0.001**; POD4 ◊ 2.26 (0.9) vs. 2.84 (0.13)	NA	NA			NA	
	Kaewborisut-sakul et al., 2023[72]	SC/IC for brain tumors	100 (obs study)	Median [IQR] total LOS; 9 [7, 12]	Median [IQR]; 0.83 [0.75, 0.96]	11 pts Sz, 36 pts ICH, 1 pt cardiovascular, 2 pts UT, 2 pts pneumonia, 1 pt DVT, 23 pts PONV, 28 pts hyperglycemia at 48 h Mortality ◊ 1 pt	NA	0%	NA			Median [IQR] total hospital costs per pt ◊ $3’799 [3’256, 4’917]	
	Khan et al., 2023[73]	SC for brain tumors	63 (int gr) vs. 457 (cont gr)	Mean (SD) total LOS; 2.6 (1.7) vs. 4.0 (3.7), ***p*** **< 0.001**	Mean (SD); 0.67 (0.27) vs. 1.2 (2.2), ***p*** **= 0.005**	1 pt postop ICH in cont gr	NA	4.8% vs. 4.0%	NA			NA	
	Sivakumar et al., 2023[74]	Brain tumors	164 (int gr) vs.?	Median total LOS at the pilot sites with ERAS ◊ 1.25	NA	NA	NA	NA	NA			Mean cost savings per pt in the pilot sites with ERAS ◊ $7’000	
	Zaed et al., 2023[75]	Brain tumors	7 (int gr) vs. 12 (cont gr)	Median total LOS; 6.3 vs. 10.5, ***p*** **= 0.001**	ICU LOS > 48 h ◊ 1 pt vs. 5 pts, ***p*** **= 0.03**	NA	NA	0 pt vs. 1 pt	NA			NA	
	Abul et al., 2024[76]	SC/IC for microvascular decompression, brain tumors, VPS, foramen magnum decompression, ETV	21 (int gr) vs. 19 (cont gr)	Total LOS was reduced by 50% in ERAS group	NA	NA	NA	NA	NA			NA	
	Staartjes et al., 2024[77]	Burr holes for cSDH or hygroma	91 (int gr) vs. 91 (cont gr)	Median [IQR]; 5 [4, 8] vs. 6 [5, 9]	NA	Any adverse events at discharge ◊ 19 pts vs. 14 pts Any adverse events at first FU ◊ 32 pts vs. 31 pts All-cause mortality up to first FU ◊ 4 pts vs. 8 pts	NA	NA	NA			NA	
	Vedicherla et al., 2025[78]	Burr holes/minicraniotomy for cSDH or subacute SDH	169 (int gr) vs. 170 (cont gr)	Median [IQR]; 7 [4, 11] vs. 9 [5, 23]	NA	Surgical complications ◊ 3 pts acute SDH, 1 pt ICH, 1 pt wound dehiscence/infection vs. 1 pt acute SDH, 1 pt ICH, 3 pts inadequate drainage, 3 pts wound dehiscence/infection; 30-day mortality ◊ 4 pts vs. 2 pts	NA	NA	NA			NA	

AC, awake craniotomy; ARDS, acute respiratory distress syndrome; CE, cerebral edema; CSF, cerebrospinal fluid; CVD, cardiovascular disease; DVT, deep vein thrombosis; ETV, endoscopic third ventriculostomy; FU, follow-up; GA, general anesthesia; GI, gastrointestinal; HA, headache; IC, infratentorial craniotomy; ICH, intracranial hemorrhage; ICU, intensive care unit; IMC, intermediate care unit; ind, induction; intraop, intraoperative; LOC, level of consciousness; main, maintenance; MI, myocardial infarction; NCCU, neurosurgical critical care unit; ns, no significant; PE, pulmonary embolism; POD, postoperative day; PONV, postoperative nausea and vomitus; postop, postoperative; pt, patient; PTSS, pain treatment satisfaction scale; SC, supratentorial craniotomy; SDH, subdural hematoma; SNB, scalp nerve block; SSI, surgical site infection; SV, supraventricular; Sz, seizure; TSS, transsphenoidal surgery; UT, urinary tract; 3-mo, 3-month

###  Same-day discharge protocols

Thirteen studies focused on same-day discharge protocols [35–47]. Of these, nine were produced by the Toronto group, using a standard protocol with selected variations based on specific indications [35–43]. The Toronto group first described [35] an outpatient awake craniotomy protocol, which was later adapted for patients requiring general anesthesia. Across these studies, same-day discharge was demonstrated to be a safe alternative to standard postoperative care in carefully selected patients, with no increase in complications or readmission rates.

###  Early discharge protocols

Three studies investigated early discharge protocols [48–50]. These demonstrated that, in selected cases, discharge within 24–48 h is feasible and does not compromise safety.

###  Avoidance of routine ICU admission protocols

Four studies analyzed protocols aiming to avoid routine ICU admission after surgery [51–54]. These consistently suggested that direct transfer to a step-down unit or regular ward is safe in patients without perioperative complications or major comorbidities, with outcomes comparable to ICU-managed cohorts.

### Awake craniotomy protocols

Two observational studies examined ERAS-based pathways specifically tailored to awake craniotomy [55, 56]. However, both were limited by small sample size and high risk of bias, preventing firm conclusions regarding safety or standardized applicability.

### Comprehensive ERAS protocols

Nineteen studies reported multimodal ERAS pathways integrating various pre-, intra-, and postoperative elements [59–78]. These protocols consistently demonstrated accelerated recovery, reduced length of stay, and lower costs, without increased complication or readmission rates. Most of these studies involved tumor resection cohorts, while two focused on chronic or subacute subdural hematoma, suggesting that ERAS principles may extend beyond oncologic neurosurgery.

The most important ERAS interventions concerning cranial surgery found in the literature either as single ERAS elements or as part of an ERAS protocol are presented in this figure. ERAS, Enhanced Recovery After Surgery; FU, follow-up; PONV, postoperative nausea and vomiting.

### Quality of evidence and recommendations

Table 3 summarizes the quality of evidence and recommendations for the most significant ERAS elements and protocols identified in cranial surgery.

**Table 3:** Level of evidence and strength of recommendation of ERAS in cranial neurosurgery

ERAS element/protocol	Recommendations	Strength of recommendation	Quality of evidence (GRADE)	Comments
Preoperative oral carbohydrate loading	The administration of preoperative oral carbohydrate loading to neurosurgical patients undergoing elective craniotomy should be considered a safe intervention to help reduce hospital stay duration	B	Moderate	This recommendation is based on only one randomized controlled trial
General anesthesia regimens	The use of a remifentanil-propofol-based anesthetic regimen, compared to a sufentanil-propofol regimen, for neurosurgical patients undergoing elective craniotomy may be considered a safe intervention with comparable costs, aimed at reducing postoperative pain	C	Low	This recommendation is based on studies involving only patients with brain tumors, with other intracranial pathologies not being represented
	Multimodal opioid-sparing anesthesia regimen for patients undergoing elective craniotomy should be considered a safe and effective strategy for reducing postoperative pain, although patient-reported satisfaction may paradoxically be lower	B	Moderate	This recommendation is based on only one randomized controlled trial
Scalp nerve block	The use of a bilateral scalp nerve block before skin incision or before emergence in neurosurgical patients undergoing elective craniotomy may be considered a safe intervention to reduce postoperative pain within the first 24 h after surgery. The use of ketamine as adjuvant to bupivacaine may be considered beneficial to improve pain control.	C	Low	This recommendation is based mainly on randomized controlled trial with small sample size and high level of inconsistency
Awake craniotomy	Awake craniotomy is not recommended for reducing postoperative complications or hospital stay duration in patients undergoing elective craniotomy	C	Very low	This recommendation is based on one interventional nonrandomized study with small sample size and serious risk of bias
	The implementation of comprehensive ERAS protocols for patients undergoing elective awake craniotomy may be considered safe; however, their impact on hospital length of stay, postoperative pain, patient satisfaction, and costs still needs to be investigated	C	Low	This recommendation is based on two interventional nonrandomized studies with small sample size and serious/critical risk of bias
Extubation in the operating room	Selective extubation of neurosurgical patients undergoing elective craniotomy in the operating room may be considered a safe intervention to reduce hospital stay duration	C	Low	This recommendation is based only on one observation study
Same-day discharge protocols	The implementation of same-day discharge protocols for patients undergoing elective craniotomy may be considered a safe intervention, with acceptable hospital readmission rate, to reduce hospital stay duration and costs	C	Low	This recommendation is based mainly on observational studies and only some nonrandomized interventional study with retrospective design
Early discharge protocols	The implementation of protocols with criteria for early discharge for patients undergoing elective craniotomy may be considered a safe intervention, with acceptable hospital readmission rate, to reduce hospital stay duration and costs	C	Low	This recommendation is based on studies involving only patients with brain tumors, with other intracranial pathologies not being represented
Avoidance of routine ICU admission protocols	The implementation of protocols that avoid routine ICU admission after elective craniotomy may be considered a safe intervention, with acceptable complication and ICU transfer rates, to reduce hospital length of stay and associated costs	C	Low	This recommendation is based mainly on observational and some nonrandomized studies with retrospective design
Early postoperative mobilization protocols	The implementation of protocols to integrate early mobilization for patients undergoing elective craniotomy should be considered a safe intervention, with acceptable hospital readmission rate, to reduce length of hospital stay and hospitalization costs.	B	Moderate	This recommendation is based on one randomized study and one large-cohort observational study
Comprehensive ERAS protocols	The implementation of comprehensive ERAS protocols for patients undergoing elective craniotomy is recommended as a safe intervention, with acceptable hospital readmission rate, to reduce hospital stay duration	A	High	This recommendation is based mainly on randomized controlled trials
	The implementation of comprehensive ERAS protocols for patients undergoing elective craniotomy should be considered as safe intervention, with acceptable hospital readmission rate, to reduce postoperative pain, increase patient satisfaction, and lower hospital stay costs	B	Moderate	This recommendation is based mainly on randomized controlled trials with moderate risk of bias and few level of inconsistency

Strength of recommendation: A = good evidence to support a recommendation for or against use; B = moderate evidence to support a recommendation for or against use; C = poor evidence to support a recommendation. ERAS, Enhanced Recovery After Surgery; GRADE, Grading of Recommendations Assessment, Development, and Evaluation; ICU, intensive care unit

## Discussion

ERAS is an increasingly researched area in neurosurgery, as evidenced by the 39 new references published after 2021, which were included in this review update, compared to the 27 studies identified prior to 2021. This expansion reflects a growing international interest in structured perioperative care protocols for cranial surgery. Consequently, the evidence base has strengthened particularly for same-day or early discharge pathways, avoidance of routine ICU admission, and nurse-driven mobilization protocols, which now include multiple prospective cohorts and standardized clinical algorithms. Furthermore, the number of comprehensive ERAS protocols combining pre-, intra-, and postoperative components has increased, and several studies now include cost analyses, protocol adherence and resource utilization metrics, which were largely absent in earlier literature. These additions indicate a maturation of ERAS concepts in neurosurgery from exploratory feasibility studies toward more systematic implementation frameworks [44, 61, 77]. 

### Beneficial pre-, intra-, and postoperative interventions in cranial surgery

Improving the quality of care can be achieved at various stages of the perioperative process. This begins with accurate preoperative patient selection for same-day surgery or early discharge protocols, using predefined selection criteria to identify suitable candidates. This risk stratification can also be assisted by clinical prediction models [79, 80]. Also, patient education by specifically trained healthcare staff about the perioperative course and what symptoms and problems to expect has been demonstrably useful. Preoperative education delivered by trained healthcare staff has also proven valuable in preparing patients for the perioperative course, including what symptoms to expect and how to manage potential complications. Interventions like preoperative carbohydrate loading have been shown to reduce postoperative insulin resistance and improve recovery [14]. Nutritional assessments, smoking and alcohol cessation, and evaluations of mental health and depression may also contribute to optimizing the patient’s preoperative condition. Intraoperatively, scalp nerve blocks have been reported satisfying results in reducing postoperative pain [81]. Other key elements, such as maintaining normothermia and euvolemia, administering standardized weight-adjusted antibiotic prophylaxis, using pneumatic stockings to prevent DVT, and providing fixed prophylaxis against PONV, are important anesthetic components of ERAS protocols, though they have not been specifically studied in cranial surgery. Moreover, minimizing intraoperative monitoring where feasible and avoiding the use of wound drains have been highlighted as beneficial practices. Postoperatively, early extubation and adherence to criteria for avoiding routine ICU admission have been found to be safe and effective. Key elements of ERAS protocols also include early mobilization, timely resumption of fluid and solid intake, minimization of urinary catheter and arterial line usage, strict fluid balance monitoring, effective PONV management, and comprehensive pain control. These measures are consistently incorporated across nearly all ERAS protocols. Lastly, the establishment of well-defined discharge criteria is essential to ERAS protocols. In same-day surgery protocols, a follow-up home visit by a nurse on the evening of surgery and the following morning has been described. Alternatively, progress can be monitored through a follow-up call by a clinical nurse to ensure patient safety and recovery.

#### Current evidence landscape of ERAS strategies in cranial surgery

Despite the growing body of literature on ERAS in cranial surgery, the overall quality of evidence remains low to moderate. Several factors contribute to this. One major issue is the high risk of bias in many of the studies, largely due to the high percentage of retrospective studies [82]. Additionally, variability in inclusion and exclusion criteria, as well as inconsistencies in outcome reporting across studies, further complicates the reliability of the data. The precision and consistency of reported outcomes are also limited by small sample sizes and the significant heterogeneity of interventions [83]. For instance, studies on scalp nerve blocks use different anesthetic agents at varying concentrations, making it difficult to compare results or draw meaningful conclusions. Furthermore, even minor variations in the individual elements of ERAS protocols can affect outcomes, adding another layer of complexity to the analysis. Another issue is the high level of indirectness in the studies, often caused by investigating slightly different patient populations [84]. This variation makes it challenging to generalize findings or apply them uniformly across neurosurgical cases. Moreover, more than half did not quantify compliance with the ERAS elements applied. As adherence is a critical driver of ERAS effectiveness, inadequate reporting of compliance substantially limits interpretability and weakens confidence in attributing outcomes to the interventions themselves.

Altogether, these factors underscore the need for more rigorous, standardized research, including larger sample sizes, prospective randomized trials, and consistent protocols based on clearly defined ERAS elements, to fully evaluate the impact of ERAS in neurosurgery.

#### Future directions

Our study provides a comprehensive overview of previous research on ERAS interventions in cranial surgery, focusing on their efficacy, safety, and their impact on postoperative complications, readmission rates, healthcare costs, and hospital and/or ICU length of stay (LOS). It is important to note that many of the existing studies were conducted by the same research groups, which may limit the generalizability of the findings. This underscores the need for replication studies in other centers to validate the results and ensure they are applicable across diverse patient populations and clinical settings. Future studies should prioritize multicenter validation across different healthcare systems, including community hospitals and resource-limited settings, to ensure broader applicability. Furthermore, specific patient populations remain underrepresented, particularly elderly patients, individuals undergoing vascular neurosurgical procedures, and patients with significant comorbidities or frailty, who may respond differently to ERAS pathways. Inclusion of these cohorts in future trials is essential to refine risk stratification and tailor ERAS protocols beyond tumor surgery. As protocol adherence is a key determinant of ERAS effectiveness, future studies should not only implement standardized pathways but also quantitatively report compliance metrics, ideally linking adherence levels with outcome improvement [7]. Additionally, the overall quality of evidence remains low to moderate, often due to small sample sizes, retrospective designs, and inconsistencies in study protocols and outcome reporting. These factors highlight the necessity for future studies that address common limitations affecting study quality. Further randomized controlled trials are essential to clarify the individual contributions of specific ERAS elements to postoperative outcome improvements. By isolating and evaluating the effects of each component and adherence to it, future studies can provide stronger evidence on which interventions are most beneficial and refine ERAS protocols for neurosurgical applications.

#### Limitations

Our study does not include a quantitative data synthesis in the form of a meta-analysis, due to the heterogeneity of the protocols applied and the variation in reported outcomes. Although our initial 2021 review was registered in PROSPERO, this updated review was not, which we acknowledge as a limitation. Future updates will be prospectively registered to enhance methodological transparency.

Despite conducting systematic search using broad terminology that aimed at including all relevant terms related to ERAS in cranial surgery, some specific interventions related to same-day or early-discharge surgery, process improvements, and cost reductions may have been overlooked if they were not framed within an ERAS study. As a result of the variability in anesthetic techniques and study designs, we were unable to draw definitive conclusions about the optimal anesthesiologic interventions for patients undergoing craniotomy within the ERAS framework. Furthermore, no specific recommendations could be made for ERAS craniotomy protocols in distinct patient populations, given the limitations of the available evidence. Finally, a substantial proportion of included studies originated from a limited number of high-volume academic centers (e.g., Toronto), which may limit validity, applicability and generalizability of the proposed recommendations to other healthcare systems, community hospitals, or resource-limited settings. However, the primary objective of our systematic review was to identify all neurosurgical studies focusing on ERAS strategies for elective craniotomies and to provide a comprehensive summary of the evidence. This overview serves as a foundational reference for future studies and highlights areas in need of further investigation to optimize ERAS protocols in neurosurgery.

## Conclusions

There has been a significant influx of evidence concerning the application of ERAS in cranial surgery. Whereas structured ERAS programs and pertinent research had already been established somewhat earlier in spinal neurosurgery, great strides have now been taken towards one of the core tenets of ERAS: the application of evidence-based elements and protocols.

This updated systematic review synthesizes available data on pre-, intra-, and postoperative ERAS interventions, as well as institution-level comprehensive protocols. Using the GRADE framework, we provide graded recommendations for ERAS components in cranial surgery. Most individual interventions are still supported by low-certainty evidence, largely due to heterogeneous methodology, small sample sizes, non-randomized study designs, and limited prospective validation. Nevertheless, several components consistently emerged as key elements within ERAS pathways for elective craniotomy: structured preoperative patient education, optimization of general anesthesia regimens, local anesthetic techniques such as SNB to improve postoperative pain control, clearly defined safety criteria for same-day or early discharge, strict selection criteria for avoiding routine ICU admission, early mobilization protocols, and the establishment of institutional evidence-based comprehensive ERAS protocols. Across multiple studies, these components were associated with reduced hospital length of stay, decreased postoperative pain, and lower healthcare costs, without an increase in readmission or complication rates.

Future research should prioritize multicenter prospective registries, adherence-monitored implementation studies, and randomized controlled trials that include broader patient populations, such as elderly individuals, patients undergoing vascular neurosurgical procedures, and those with significant comorbidities or frailty. Standardized reporting of protocol fidelity and outcome definitions will also be essential to identify which ERAS components provide the greatest incremental benefit. Taken together, ERAS as a concept has the potential of exhibiting real-world impact in most patients undergoing routine cranial surgery by improving the preoperative patient state, limiting the deleterious effects of the surgical procedure itself, and boosting the recovery process in the early postoperative phase.

## Supplementary Information

Below is the link to the electronic supplementary material.

Supplementary Material 1

## Data Availability

No datasets were generated or analysed during the current study.
