# Supplementary material for: Level of evidence of enhanced recovery after surgery (ERAS) strategies for elective craniotomy: An updated systematic review
Source: Neurosurg Rev. 2026 Jan 13;49(1):122. doi: 10.1007/s10143-025-03961-9 (PMC13653864; doi:10.1007/s10143-025-03961-9)
Supplement: Supplementary file 1 [file 10143_2025_3961_MOESM1_ESM.docx]

# **Supplemental Methods 1: Search Strategy**

Exact search strategies stratified by database and number of results.

Search terms: fast track; ERAS; enhanced recovery; early recovery; craniotomy.

Exceptions: None.

Strategy: (ERAS OR fast track OR enhanced recovery OR early recovery) AND (craniotomy).

PubMed/MEDLINE: 121; Embase: 239; Scopus: 23; Cochrane Library: 32; Other sources: 7.

# **SUPPLEMENTAL TABLES**

Supplemental Table 1. Study characteristics of included study (alphabetical order).

| **Authors & Year** | **Study type** | **Study design** | **ERAS** | **Indication** | **No. of Pts** | **Anesthesia** | **Age, years** | **Sex (M/F)** | **Adherence** | **Risk of Bias†** |
| --- | --- | --- | --- | --- | --- | --- | --- | --- | --- | --- |
| Abhinav et al., 2023^1^ | Int | RCT | Protocol | SC & IC for brain tumor not further specified | 128 (int gr) *vs* 130 (cont gr) | GA | Mean; 45 *vs* 46 | 77/51 *vs* 72/58 | NA | Low |
| Abul et al., 2024^2^ | Int | RS-PD w/ HistC | Protocol | SC & IC for microvascular decompression (n=19); brain tumors not further specified (n=18); VPS (n=1); foramen magnum decompression (n=2); ETV (n=2) | 21 (int gr) *vs* 19 (cont gr) | GA | Mean; 53 *vs* 60 | NA | NA | Critical |
| Akcil et al., 2017^3^ | Int | RCT | Element | IC for brain tumors not further specified (n=23); Arnold-Chiari malformation (n=22) | 15 (int gr1) *vs* 15 (int gr2) *vs* 15 (cont gr) | GA | Mean (SD); 41 (16) *vs* 38 (11) *vs* 40 (18) | 7/8 *vs* 4/11 *vs* 4/11 | NA | High |
| Au et al., 2016^4^ | Obs | RS-PD | Protocol | SC for brain tumors not further specified | 44 | GA | Mean; 47 | 19/25 | 86% | Moderate |
| Azghadi et al., 2025^5^ | Int | RS-PD w/ HistC | Protocol | NA | 23 (int gr) *vs* 38 (cont gr) | AC | Mean (SD); 53 (17) *vs* 53 (14) | 16/7 *vs* 27/11 | NA | Serious |
| Bala et al., 2022^6^ | Int | RCT | Element | SC for brain tumors 🡪 meningioma (n=25); glioma (n=13); orbital mass (n=2); colloidal cyst (n=5); others (n=5) | 25 (int gr) *vs* 25 (cont gr) | GA | Mean (SD); 44 (14) *vs* 42 (15) | 13/12 *vs* 12/13 | 100% | High |
| Bastola et al., 2015^7^ | Int | RT | Element | SC for brain tumors 🡪 glioma (n=57); meningioma (n=14); others (n=4) | 25 (int gr1) *vs* 25 (int gr2) *vs* 25 (int gr3) | GA | Mean (SD); 38 (13) *vs* 40 (13) *vs* 43 (13) | 18/7 *vs* 18/7 *vs* 17/8 | NA | High |
| Beauregard and Friedman, 2003^8^ | Obs | RS-PD | Element | SC & IC for brain tumors not further specified; microvascular decompression; Arnold-Chiari malformation | 430 | GA | Mean (range); 54 (19, 78) | 183/246 | NA | Moderate |
| Bernstein et al., 2001^9^ | Obs | PS | Protocol | SC for brain tumors 🡪 metastasis (n=21); glioma (n=19); others (n=9) | 46 | AC | Mean (range); 52 (18, 78) | 21/25 | 89% | Moderate |
| Bhagat et al., 2008^10^ | Int | RT | Element | SC for brain tumors 🡪 meningioma (n=28); glioma (n=78); others (n=30) | 47 (int gr1) *vs* 45 (int gr2) *vs* 44 (int gr3) | GA | Mean (SD); 40 (11) *vs* 32 (12) *vs* 37 (12) | 31/16 *vs* 29/16 *vs* 26/18 | NA | Some concerns |
| Bojaxhi et al., 2021^11^ | Int | RS-PD | Element | SC for brain tumors 🡪 meningioma (n=2); glioma (n=78); metastasis (n=11) | 56 (int gr) *vs* 35 (cont gr) | AC & GA | Mean (SD); 49 (4) *vs* 57 (6) | 30/26 *vs* 19/16 | NA | Moderate |
| Boulton and Bernstein, 2008^12^ | Obs | PS | Protocol | SC for brain tumors not further specified | 262 | AC | NA | NA | 94% | Moderate |
| Carella et al., 2021^13^ | Int | RCT | Element | SC for brain tumors not further specified (n=53); unruptured aneurysm (n=6); AVM (n=1) | 30 (int gr) *vs* 30 (cont gr) | GA | Mean (range); 57 (22, 78) *vs* 57 (30, 78) | 13/17 *vs* 13/17 | 100% | High |
| Cata et al., 2011^14^ | Obs | RS | Element | IC for brain tumors not further specified (n=119); vascular pathologies not further specified (n=26) | 145 | GA | Mean (range); 52 (40, 65) | 70/75 | 82% | Serious |
| Chen et al., 2021^15^ | Obs | RS-PD | Protocol | Craniotomy for meningioma (n=4); glioma (n=7); acoustic neuroma (n=1); hemangioblastoma (n=1); epidermoid cyst (n=1); microvascular decompression (n=6) | 20 | AC | Mean (SD); 50 (18) | 10/10 | 100% | Critical |
| Chen et al., 2025^16^ | Int | RCT | Protocol | Glioma (n=115) | 59 (int gr) *vs* 56 (cont gr) | GA | Mean (SD); 53 (12) *vs* 52 (14) | 38/21 *vs* 33/23 | NA | Some concerns |
| Choi et al., 2023^17^ | Int | RCT | Element | SC for unruptured aneurysms | 27 (int gr) *vs* 25 (cont gr) | GA | Mean (SD); 59 (9) *vs* 63 (10) | 5/22 *vs* 4/21 | 100% | High |
| Djian et al., 2006^18^ | Int | RCT | Element | SC for brain tumors 🡪 meningioma (n=20); glioma (n=18); metastasis (n=12); others (n=10) | 29 (int gr1) *vs* 31 (int gr2) | GA | Mean (SD); 47 (12) *vs* 55 (9) | 11/18 *vs* 12/19 | NA | High |
| Elayat et al., 2021^19^ | Int | PS | Protocol | SC for brain tumors 🡪 meningioma (n=41); glioma (n=19); others (n=10) | 35 (int gr) *vs* 35 (cont gr) | GA | Mean (SD); 41 (14) *vs* 47 (14) | 14/21 *vs* 16/19 | 100% | Serious |
| Feng et al., 2021^20^ | Int | RS-PD | Protocol | SC & IC for brain tumors 🡪 meningioma (n=31); glioma (n=31); others (n=38) | 50 (int gr) *vs* 50 (cont gr) | GA | Mean (SD); 48 (12) *vs* 48 (15) | 21/29 *vs* 23/27 | 100% | Moderate |
| Gerlach et al., 2003^21^ | Int | RT | Element | SC brain tumors not further specified | 18 (int gr1) *vs* 18 (int gr2) | GA | Mean (SD); 53 (13) *vs* 47 (14) | 6/11 *vs* 5/13 | NA | High |
| Goettel et al., 2014^22^ | Obs | RS-PD | Protocol | SC for unruptured aneurysms | 25 | GA | Mean (SD); 54 (9) | 11/14 | 68% | Serious |
| Grundy et al., 2008^23^ | Obs | PS | Protocol | SC for brain tumors 🡪 glioma (n=31); primitive neuroectodermal tumor (n=1); dysembryoplastic neuroepithelial tumor (n=1); PCNSL (n=2); metastasis (n=5); others (n=1) | 41 | AC & GA | Mean (range); 51 (18, 82) | 19/22 | 88% | Critical |
| Gurses aet al., 2024^24^ | Obs | RS-PD | Protocol | SC for brain tumors 🡪 metastasis (n=45), meningioma (n=42), GBM (n=25), LGG (n=21); aqueductal stenosis (n=11), lymphoma (n=10); others (n=48) | 202 | GA & AC | Mean (SD); 57 (16) | 85/117 | 88.6% | Moderate |
| Hoffman et al., 2023^25^ | Int | RS-PD w/ HistC | Protocol | SC for brain tumors 🡪 glioma (n=121); meningioma (n=57); metastasis (n=142); others (n=75) | 202 (int gr) *vs* 193 (cont gr) | GA | Mean (SD); 58 (14) *vs* 60 (15) | 96/106 *vs* 87/106 | 97% | Moderate |
| Hussein et al., 2020 | Int | PS | Element | SC for brain tumors not further specified | 15 (int gr) *vs* 15 (cont gr) | GA | Mean (SD) 42 (13) *vs* 42 (11) | 10/5 *vs* 11/4 | NA | Critical |
| Kadian aet al., 2025^26^ | Int | RCT | Element | SC for for various intracranial lesions | 33 (int gr1) *vs* 32 (int gr2) *vs* 32 (cont gr) | GA | Mean (SD); 39 (16) *vs* 39 (14) *vs* 40 (13) | 20/13 *vs* 17/15 *vs* 20/12 | NA | Low |
| Kaewborisutsakul et al., 2023^27^ | Obs | RS-PD | Protocol | SC & IC for brain tumors 🡪 meningioma (n=50); CPA tumors (n=4); craniopharyngioma (n=2); germinoma (n=1); metastasis (n=14); glioma (n=29) | 100 | GA | Median (range) *vs* 53 (15, 86) | 31/69 | 55% >8 ERAS protocol items | Moderate |
| Khan et al., 2023^28^ | Int | PS w/ HistC | Protocol | SC for brain tumors 🡪 glioma (n=211); metastasis (n=25); extra-axial primary tumors (n=263); lymphoma (n=3); others (n=18) | 63 (int gr) *vs* 457 (cont gr) | GA | Mean (SD) 51 (15) *vs* 53 (14) | 33/30 *vs* 178/279 | NA | Moderate |
| Levy et al., 2023^29^ | Int | RS-PD | Protocol | SC for brain tumors 🡪 meningioma (n=14); osteoid osteoma (n=4); metastasis (n=28); glioma (n=33); cavernoma (n=3); benign hemangioma (n=3); abscess/infectious process (n=4); inflammatory process (n=1); PNET (n=1) | 31 (int gr) *vs* 59 (cont gr) | GA | Median (range); 61 (19, 78) *vs* 59 (21, 85) | 5/26 *vs* 23/36 | 90% | Serious |
| Liu et al., 2019^30^ | Int | RCT | Protocol | SC & IC for meningioma (n=68); acoustic neuroma (n=16); CPA cholesteatoma (n=14); glioma (n=31); trigeminal neuralgia (n=6); cavernoma (n=5) | 70 (int gr) *vs* 70 (cont gr) | GA | Mean (range); 51 (19, 67) *vs* 49 (18, 65) | 22/48 *vs* 26/44 | 100% | Low |
| Liu et al., 2019^31^ | Int | RCT | Element | SC & IC for meningioma (n=57); acoustic neuroma (n=15); CPA cholesteatoma (n=10); glioma (n=27); trigeminal neuralgia (n=6); cavernoma (n=5) | 58 (int gr) *vs* 62 (cont gr) | GA | <40 y 34 pts *vs* 34 pts; 40-65 y 24 pts *vs* 28 pts | 17/41 *vs* 22/40 | 100% | Low |
| Liu et al., 2020^32^ | Int | RCT | Protocol | SC & IC for glioma | 36 (int gr) *vs* 29 (cont gr) | GA | <40 y 12 pts *vs* 8 pts; 40-65 y 24 pts *vs* 21 pts | 19/17 *vs* 16/13 | NA | High |
| Ma et al., 2016^33^ | Obs | RS-PD | Protocol | SC for brain tumors 🡪 meningioma (n=3); glioma (n=53); hemangioblastoma (n=1); metastasis (n=18) | 75 | AC & GA | Mean (range); 52 (24, 75) | 39/36 | NA | Moderate |
| McLaughlin et al., 2014^34^ | Int | RS-PD w/ HistC | Protocol | IC for trigeminal neuralgia (n=28); hemifacial spasm (n=16) | 25 (int gr1) *vs* 7 (int gr2) *vs* 12 (cont gr) | GA | NA | NA | NA | Moderate |
| McLaughlin et al., 2014^35^ | Int | RS-PD w/ HistC | Protocol | IC for trigeminal neuralgia (n=31); hemifacial spasm (n=18) | 29 (int gr) *vs* 20 (cont gr) | GA | Mean (range); 56 (32, 83) *vs* 57 (26, 57) | 6/14 *vs* 7/22 | NA | Moderate |
| Nassiri et al., 2019^36^ | Obs | RS-PD | Protocol | SC for brain tumors 🡪 meningioma (n=1); metastasis (n=5); glioma (n=22); others (n=1) | 29 | AC | Mean; 55 | 21/8 | NA | Moderate |
| Negm et al., 2025^37^ | Int | RCT | Element | SC for various intracranial lesions | 30 (int gr) *vs* 30 (cont gr) | GA | Mean (SD); 41 (11) *vs* 46 (14) | 18/12 *vs* 14/16 | NA | Low |
| Neville et al., 2020^38^ | Int | RS-PD w/ HistC | Protocol | SC & IC for brain tumors 🡪 meningioma (n=1); glioma (n=36); metastasis (n=28); inflammatory process (n=1) | 34 (int gr) *vs* 32 (cont gr) | AC & GA | Mean (SD); 54 (13) *vs* 49 (15) | 21/13 *vs* 18/14 | NA | Serious |
| Nijs et al., 2023^39^ | Obs | RS-PD | Protocol | SC for brain tumors not further specified | 630 | AC & GA | Mean (SD); 51 (15) | 319/311 | 91% | Low |
| Peláez-Sanchez et al., 2023^40^ | Int | RS-PD | Protocol | SC for brain tumors 🡪 meningioma (n=10); glioma (n=31); metastasis (n=21); others (n=15) | 18 (int gr) *vs* 59 (cont gr) | GA | Mean (SD); 66 (8) *vs* 66 (12) | 9/9 *vs* 25/34 | 89% | Serious |
| Peláez-Sanchez et al., 2025^41^ | Int | RS-PD | Protocol | SC for brain tumors 🡪 HGG (n=24), LGG (n=1), meningioma (n=9), metastasis (n=15), others (n=7) | 18 (int gr) *vs* 38 (cont gr) | GA | Mean (SD); 66 (8) *vs* 65 (12) | 9/9 *vs* 17/21 | 89% | Serious |
| Perez-Vega et al., 2023^42^ | Obs | RS-PD | Protocol | Craniotomy for brain tumors 🡪 meningioma (n=20); glioma (n=17); metastasis (n=7); others (n=13) | 57 | AC & GA | Mean (range); 56 (25, 83) | 23/34 | 97% | Moderate |
| Purzner et al., 2011^43^ | Obs | RS-PD | Protocol | SC for brain tumors not further specified | 401 | AC | NA | NA | 93% | Moderate |
| Qu et al., 2020^44^ | Int | RCT | Protocol | SC & IC for brain tumors 🡪 meningioma (n=68); acoustic neuroma (n=14); CPA cholesteatoma (n=14); glioma (n=31) | 64 (int gr) *vs* 65 (cont gr) | GA | <40 y 38 pts *vs* 36 pts; 40-65 y 26 pts *vs* 29 pts | 21/43 *vs* 24/41 | NA | Some concerns |
| Rajan et al., 2016^45^ | Int | RCT | Element | SC, IC & TSS for brain tumors not further specified | 71 (int gr) *vs* 71 (cont gr) | GA | Mean (SD); 56 (14) *vs* 55 (14) | 38/33 *vs* 34/37 | 98% | Some concerns |
| Rigamonti et al., 2020^46^ | Int | RCT | Element | SC for brain tumors not further specified (n=82); unruptured aneurysms (n=5); AVM (n=1); cavernoma (n=1) | 44 (int gr) *vs* 45 (cont gr) | GA | Mean (SD); 54 (15) *vs* 55 (15) | 16/28 *vs* 24/21 | 93% | Some concerns |
| Sarmento et al., 2020^47^ | Int | RS-PD w/ HistC | Element | SC for mesial temporal lobe epilepsy | 43 (int gr) *vs* 30 (cont gr) | GA | Mean (SD); 31 (14) *vs* 35 (8) | 20/23 *vs* 13/17 | NA | Serious |
| Sivakumar et al., 2023^48^ | Int | PS | Protocol | Craniotomy for brain tumors not further specified | 164 (int gr) *vs*? | GA | NA | NA | NA | Serious |
| Staartjes et al., 2024^49^ | Int | RS-PD w/ HistC | Protocol | Burr holes for cSDH or hygroma | 91 (int gr) *vs* 91 (cont gr) | AC & GA | Median [IQR]; 76 [67, 82] *vs* 77 [66, 84] | 67/24 *vs* 66/25 | NA | Moderate |
| Sughrue et al., 2015^50^ | Obs | RS-PD | Protocol | SC & IC for brain tumors 🡪 glioma (n=185); meningioma (n=44); metastasis (n=27); acoustic neuroma (n=21); intraventricular tumors (n=13); others (n=23) | 213 | GA | Mean (SD); 44 (17) | 102/111 | NA | Serious |
| Ter Laan et al., 2020^51^ | Int | RS-PD w/ HistC | Protocol | SC for brain tumors not further specified | 109 (int gr) *vs* 107 (cont gr) | GA | Mean; 55 *vs* 56 | 57/52 *vs* 50/57 | NA | Moderate |
| Todd et al., 1993^52^ | Int | RT | Element | SC for brain tumors not further specified | 40 (int gr1) *vs* 40 (int gr2) *vs* 40 (int gr3) | GA | Mean (SD); 50 (12) *vs* 52 (15) *vs* 50 (17) | 16/24 *vs* 20/20 *vs* 25/16 | 100% | High |
| Uchida et al., 2012^53^ | Int | RS-PD | Element | Craniotomy for brain tumors not further specified | 936 (int gr) *vs* 936 (cont gr) | GA | Mean (SD); 55 (18) *vs* 55 (17) | 427/509 *vs* 427/509 | NA | Moderate |
| Vedicherla et al., 2025^54^ | Int | RS-PD w/ HistC | Protocol | Burr holes/minicraniotomy for cSDH or subacute SDH | 169 (int gr) *vs* 170 (cont gr) | GA & LA | Mean (SD); 74 (10) *vs* 74 (11) | 136/33 *vs* 123/47 | NA | Moderate |
| Venkatraghavan et al., 2016^55^ | Obs | RS-PD | Protocol | SC for brain tumors 🡪 glioma (n=72); metastasis (n=49); meningioma (n=20); others (n=36) | 198 | AC & GA | Mean (SD); 50 (15) | 112/86 | 88% | Moderate |
| Wagner et al., 2025^56^ | Obs | RS-PD | Protocol | SC & IC for brain tumors and vascular lesions | 5638 | GA | Mean (SD); 54 (16) | 2486/3152 | 98.5% | Moderate |
| Wang et al., 2018^57^ | Int | RCT | Protocol | SC & IC for meningioma (n=68); acoustic neuroma (n=16); CPA cholesteatoma (n=14); glioma (n=31); trigeminal neuralgia (n=6); cavernoma (n=5) | 70 (int gr) *vs* 70 (cont gr) | GA | Mean (range); 51 (19, 67) *vs* 49 (18, 65) | 22/48 *vs* 26/44 | 100% | Low |
| Wang et al., 2022^58^ | Int | RCT | Protocol | SC for glioma | 80 (int gr) *vs* 71 (cont gr) | AC & GA | Mean (SD); 52 (12) *vs* 49 (12) | 52/28 *vs* 38/33 | 98% | Low |
| Wang et al., 2022^59^ | Int | RCT | Protocol | SC & IC for trigeminal neuralgia (n=50); hemifacial spasm (n=52); meningioma (n=17); glioma (n=15); cholesteatoma (n=5); hemangioma (n=6); acoustic neuroma (n=6) | 76 (int gr) *vs* 75 (cont gr) | GA | Mean (SD); 53 (11) *vs* 51 (10) | 30/46 *vs* 32/43 | NA | Low |
| Wu et al., 2021^60^ | Int | RCT | Protocol | SC for brain tumors not further specified | 76 (int gr) *vs* 75 (cont gr) | GA | NA | NA | NA | Some concerns |
| Yakar et al., 2023^61^ | Obs | RS-PD | Protocol | SC for unruptured aneurysms | 38 institutions | GA | NA | NA | Median (min, max); 66% (39, 91) | Serious |
| Yang et al., 2019^62^ | Int | RCT | Element | SC for unruptured aneurysms | 19 (int gr1) *vs* 19 (int gr2) *vs* 19 (cont gr) | GA | Mean (SD); 56 (5) *vs* 55 (6) *vs* 54 (7) | 6/12 *vs* 4/12 *vs* 4/13 | 89% | Some concerns |
| Yang et al., 2020^63^ | Int | RCT | Element | NA | 21 (int gr1) *vs* 20 (int gr2) *vs* 22 (int gr3) *vs* 22 (cont gr) | GA | Mean (SD); 43 (12) *vs* 43 (12) *vs* 46 (11) *vs* 44 (13) | 11/10 *vs* 12/8 *vs* 11/11 *vs* 7/15 | 100% | High |
| Zaed et al., 2023^64^ | Int | RS-PD w/ HistC | Protocol | Craniotomy for glioma (n=19) | 7 (int gr) *vs* 12 (cont gr) | GA | Mean (range); 77 (75, 81) *vs* 76 (75, 83) | 2/5 *vs* 5/7 | NA | Critical |
| Zarrin et al., 2025^65^ | Obs | RS-PD | Protocol | Craniotomy for brain tumors 🡪 pituitary tumors (n=466), glioma (n=217), meningioma (n=186), metastasis (n=81), other (n=109) | 1059 | GA | Mean (SD); 54 (16) | 150/139 | 78.7% | Moderate |

AC, awake craniotomy; CPA, cerebellopontine angle; cont, control; ERAS, enhanced recovery after surgery; ETV, endoscopic third ventriculostomy; GA, general anesthesia; HistC, historical cohort; IC, infratentorial craniotomy; int, interventional; LA, local anesthesia; LGG, low-grade glioma; NA, not applicable; obs, observational; PCNSL, primary central nervous system lymphoma; PS, prospective study; Pts, patients; RCT, randomized controlled trial; RS, retrospective study; RS-PD, retrospective study on prospectively collected data; RT, randomized trial; SC, supratentorial craniotomy; SD, standard deviation; TSS, transsphenoidal surgery; VPS, ventriculoperitoneal shunt; w/, with; w/o, without.

†The risk of bias of each included article was assessed using either the Cochrane Risk of Bias In Non-Randomized Studies of Interventions (ROBINS-I) tool or the Revised Cochrane Risk of Bias Tool for Randomized Trials (RoB2). According to the ROBINS-I tool risk of bias is graded as low/moderate/serious/critical; according to RoB2 tool risk of bias is graded as low/some concerns/high.

Supplemental Table 2. Description of cranial ERAS elements.

| **Element description** | | **Authors & Year** | **Specific inclusion criteria** | **Specific exclusion criteria** |
| --- | --- | --- | --- | --- |
| *Preoperative* | |  |  |  |
|  | Oral food intake |  |  |  |
|  | Oral carbohydrate loading *vs* standard fasting | Liu et al., 2019^31^ | Pts 18-65 yrs w/ single intracranial lesion, eligible for elective craniotomy | Intracranial trauma, pathology requiring emergency op, preop disturbance of consciousness, significant cognitive impairment unable to cooperate, confounding condition (e.g., pregnancy) or disease that could potentially impact postoperative recovery (e.g., paralysis, spinal deformity), diabetes or a preoperative fasting glucose greater than 7 mmol/L, using steroids or immunosuppressants, history of abnormal gastric emptying or intestinal obstruction. |
| *Intraoperative* | |  |  |  |
|  | Comparison of GA regimens: |  |  |  |
|  | (A) Propofol (ind) + fentanyl-propofol (main) *vs* thiopental (ind) + isoflurane-nitrous oxide (main) *vs* thiopental (ind) + fentanyl-nitrous oxide-isoflurane (main) | Todd et al., 1993^52^ | Pts 18-75 yrs w/ supratentorial mass lesion treated w/ elective craniotomy for resection, alert and oriented, able to sign informed consent, ASA II-III, no anaesthesiologic contraindications for the administration of the three anaesthetic regimens | Known aneurysms, arteriovenous malformations, or posterior fossa tumors; relevant comorbidities (e.g. severe ischemic heart disease, renal or hepatic dysfunction); need for postoperative sedation and mechanical intubation; craniotomy w/in the last 6 months |
|  | (B) Remifentanil-propofol *vs* sufentanil-propofol | Gerlach et al., 2003^21^; Djian et al., 2006^18^ | Gerlach et al.: Pts 18-70 yrs treated w/ supratentorial tumor resection, ASA I-III; alert and oriented with respect of time, place, and person before operation  Djian et al.: Pts 19-75 yrs treated w/ supratentorial neurosurgical procedure, surgery max. duration of 480 min, ASA I-III | Gerlach et al.: relevant comorbidities (e.g., ischemic heart disease, liver disorder or renal disease), previously known allergic reactions  Djian et al.: altered consciousness, relevant comorbidities (e.g., renal or hepatic dysfunction, severe chronic respiratory disease); possible postop sedation and mechanical ventilation required |
|  | (C) Propofol *vs* fentanyl *vs* isoflurane | Bhagat et al., 2008^10^ | Pts 20-60 yrs treated w/ elective craniotomy for supratentorial tumors | Altered consciousness, relevant comorbidities (e.g., ischemic and/or congestive heart disease, hypertension, chronic obstructive pulmonary disease, diabetes mellitus, hepatic and renal dysfunction), postop ventilation required |
|  | (D) Remifentanil-fentanyl *vs* fentanyl alone | Uchida et al., 2012^53^ | Pts treated w/ elective brain tumor resection with open craniot2omy under GA | Altered consciousness, relevant comorbidities (e.g., cerebrovascular disease, chronic renal failure, liver cirrhosis) |
|  | (E) Propofol *vs* sevoflurane *vs* desflurane | Bastola et al., 2015^7^ | Pts 20-60 yrs, ASA I-II | Altered consciousness, relevant comorbidities (e.g., ischaemic and/or congestive heart disease, hypertension, diabetes mellitus, chronic obstructive pulmonary disease, hepatic and renal dysfunction) |
|  | (F) Dexmedetomidine *vs* remifentanil | Rajan et al., 2016^45^ | Pts treated w/ elective surgical excision of a brain tumor (open supra- or infratentorial craniotomy or transsphenoidal approach) under GA | BMI > 45 kg/m^2^, relevant comorbidities (hypertension, cardiac conduction defects), chronic pain requiring opioid analgesia, reported allergy to either study drugs, AC or craniotomies requiring intraop magnetic resonance scanning |
|  | (G) Multimodal opioid-sparing analgesia (dexmedetomidine infusion + SNB w/ bupivacaine before emergence) *vs* conventional opioid-based analgesia | Negm et al., 2025^37^ | Age 18–65 years, ASA physical status I–II, short-term steroid use (<5 days) or antiepileptic therapy ≤2 weeks allowed | GCS <14, neurological impairment, uncontrolled hypertension, renal/hepatic dysfunction, bronchial asthma, long-term steroid, antiepileptic, antidepressant, analgesic, or neuropathic agent use, psychiatric disorders, drug dependence, allergy to study drugs, extensive surgery (>6 h), massive hemorrhage, or need for postoperative ventilation, previous craniotomy |
|  | AC *vs* GA: |  |  |  |
|  | AC consisted in dexmedetomidine-propofol infusion + SNB + injection of ropivocaine-epinephrine at pine entry sites and subdural; GA consisted in induction w/ propofol-fentanyl-rocuronium/vecuronium + maintenance w/ propofol-dexmedetomidine-fentanyl | Bojaxhi et al., 2021^11^ | Pts ≥ 18 yrs w/ single, unilateral lesion treated w/ pterional or frontotemporal craniotomy | Preop opioid use, history of chronic pain, allergies to associated medications, major comorbidities (i.e. ASA IV), urgent/emergent cases |
|  | Bilateral SNB before skin incision: |  |  |  |
|  | (A) SNB w/ bupivacaine *vs* local anesthesia w/ bupivacaine *vs* remifentanil alone | Akcil et al., 2017^3^ | Pts 18-70 yrs, ASA I-III | Allergy to bupivacaine or opioids, relevant comorbidities (e.g., chronic hypertension, coronary artery disease, arrhythmia, coagulopathy, cerebrovascular disease), raised intracranial pressure, trigeminal neuralgia and previous craniotomy |
|  | (B) SNB w/ ropivocaine *vs* local anesthesia w/ ropivocaine *vs* remifentanil alone | Yang et al., 2019^62^ | Pts 18-65 yrs treated w/ elective aneurysm clipping, ASA I-II | Altered consciousness, difficult surgical anatomy or multiple/giant aneurysms, previous craniotomy incision, history of allergy to opioids or local anesthetics, history of drug dependence and alcohol abuse, unable to understand VAS or communicate, scheduled to be sedated postop |
|  | (C) SNB w/ ropivacaine (different concentrations) *vs* sham SNB | Yang et al., 2020^63^ | Pts 18-60 yrs treated w/ elective craniotomy that required GA, ASA I-II, BMI 18-30 kg/m^2^ | Altered consciousness, unable to understand or use VAS, allergic to local anesthetics, history of opioid dependence, coagulopathy, scalp infection, pregnancy, previous craniotomy, complications of surgery, aphasia, unconsciousness, postop mechanical ventilation, duration of surgery > 6 hr |
|  | (D) SNB w/ bupivacaine *vs* sham SNB | Carella et al., 2021^13^ | Pts 18-y5 yrs treated w/ elective supratentorial surgery, ASA I-III, receive GA, surgery planned in the supine position with an estimated time length of 90-360 min | Contraindications to SNB (e.g. known allergy to used medications or local infection), BMI > 35 kg/m², emergency craniotomies, chronic pain (persistent or recurrent pain lasting > 3 months) or fibromyalgia, drug addiction (illicit substances and opioid regular use, chronic alcohol abuse), treatment with corticosteroids > 6 months, uncontrolled systemic arterial hypertension, relevant comorbidities (e.g., severe kidney or liver diseases, mental disorders or serious neurological diseases, cardiomyopathies), major intraop hemorrhage |
|  | (E) SNB w/ bupivacaine & ketamine *vs* bupivacaine & magnesium sulfate *vs* bupivacaine alone | Kadian et al., 2025^26^ | Age 18–65 years, ASA physical status I–III, GCS 13–15 | Local infection at block site, coagulation disorders, psychotic disorders or substance abuse, preop cognitive impairment or chronic headache, long-term analgesic use, allergies to study drugs, uncontrolled hypertension, diabetes, or cardiac disease |
|  | Bilateral SNB before emergence: |  |  |  |
|  | (A) SNB w/ bupivocaine-lidocaine-epinephrine *vs* fentanyl alone | Hussein et al., 2020 | Pts 21-60 yrs w/ supratentorial tumor treated w/ craniotomy under general anaesthesia, ASA I-II, BMI < 35 kg/m² | Altered consciousness, huge tumour with marked midline shift, incision extending beyond the areas covered by SNB, local anaesthetic infiltration preop along the planned scalp incision or postop into the wound edges only, previous craniotomy, chronic use of analgesics, drug dependence, uncontrolled hypertension, duration of surgery > 6 hr, pt needing postoperative ventilatory support, any complications during the procedure |
|  | (B) SNB w/ bupivocaine-epinephrine *vs* sham SNB | Rigamonti et al., 2020^46^ | Pts ≥ 18 yrs, treated w/ elective supratentorial craniotomy, ASA I–III | Contraindications the use of the anesthesia maintenance protocol of the study, potential difficulties to discern postop pain specifically related to the surgery itself |
|  | (C) SNB w/ ropivacaine-epinephrine *vs* sham SNB | Choi et al., 2023^17^ | Pts 20–79 yrs w/ unruptured intracranial aneurysms treated w/ elective single keyhole minicraniotomy for clipping at single academic tertiary hospital | Symptomatic aneurysm, allergies to local anesthetics or analgesics, chronic use of opioids, history of craniotomy or scalp incision, other diseases, ASA ≥ III, unable to communicate with their healthcare provider or complete a written version of the QoR-40K |
|  | Minimally invasive craniotomy |  |  |  |
|  | Nummular craniotomy of the temporal bone *vs* conventional frontotemporal craniotomy | Sarmento et al., 2020^47^ | Pts w/ refractory mesial temporal lobe epilepsy and unilateral hippocampal sclerosis | NA |
|  | Anesthetic injection in the subdural drain |  |  |  |
|  | Injection of ropivacaine in the subdural drain at the end of surgery *vs* sham injection | Bala et al., 2022^6^ | Pts 18–65 yrs treated w/ elective supratentorial craniotomy under general anaesthesia, ASA I-III | Altered consiousness, unable to communicate or comprehend, allergy to local anaesthetics, previous craniotomy, history of drug/alcohol abuse, posted for aneurysm clipping or planned for postop ventilator support |
| *Postoperative* | |  |  |  |
|  | Extubation timepoint |  |  |  |
|  | Extubation in OR | Cata et al., 2011^14^ | Pts w/ primary or secondary tumors, vascular malformations, and fourth ventricle cysts treated w/ surgery | NA |
|  | Floor disposition |  |  |  |
|  | ICU *vs* ICM *vs* floor | Beauregard and Friedman, 2003^8^ | Pts ≥ 18 yrs w/ brain tumor, pituitary adenoma, trigeminal neuralgia, hemifacial spasm, and Chiari malformation treated w/ elective craniotomies | Craniotomies for aneurysm, AVM, or epilepsy |

AC, awake craniotomy; GA, general anesthesia; ICU, intensive care unit; IMC, intermediate care unit; ind, induction; main, maintenance; OR, operating room; SNB, scalp nerve block; w/, with; w/o, without; yrs, years.

Supplemental Table 3. Description of cranial ERAS protocols.

| **Protocol & Authors** | **Summary of Protocol** | | |
| --- | --- | --- | --- |
| *Same-Day Discharge Protocols* | | |  |
| (A) Toronto protocol (Bernstein et al., 2001^9^; Boulton & Bernstein, 2008^12^; Grundy et al., 2008^23^; Purzner et al., 2011^43^; Goettel et al., 2014^22^; Au et al., 2016^4^; Venkatraghavan et al., 2016^55^; Nassiri et al., 2019^36^; Nijs et al., 2023^39^) | | | |
| Treated pathologies | Different types of brain tumors; unruptured aneurysms of the anterior circulation. | | |
| Eligibility | Pts living relatively w/ caregiver capable of recognizing a change in neurological status and relatively close to the hospital. Exclusion: preexisting inpatient status, significant medical comorbidity requiring hospital management, significant existing neurological deficit altering mobility, preference to remain in hospital overnight, surgery not expected to be completed early enough to allow sufficient time for requisite observation period. | | |
| Preop | Presurgical office visit: pt & caregiver education about expected course of events & potential complications of the procedure. Informed consent: information pamphlet & contact information for the neurooncology nurse practitioner to address further doubts. Surgeon availability for additional visits as required. Early admission the day of surgery, preop MRI. | | |
| Intraop | **AC** 🡪 IV line access; no routine use of arterial lines, urinary catheters, or central venous pressure lines. Nasal prongs for oxygen, standard monitoring (ECG, pulse oximetry & BP), pin-site local anesthesia w/ xylocaine injection, frameless neuronavigation registration. Prophylactic antibiotics &, usually, steroids; AEDs not routine unless already on medication for Sz; mannitol not administered routinely; scalp anesthesia: bupivacaine w/ epinephrine. Anesthetics: short-acting IV agents for sedation: propofol, midazolam, fentanyl, or remifentanil.  **GA** 🡪 monitoring w/ ECG, end-tidal capnography, pulse oximetry, & arterial catheterization; somatosensory evoked potential monitoring. Prophylactic antibiotics & steroids prior to the skin incision & AEDs if pt has previously had a Sz. Induction: IV bolus of propofol & fentanyl, NMB w/ rocuronium administration. Maintenance: inhalational anesthetic agent (either sevoflurane or desflurane) & either intermittent boluses of fentanyl or IV remifentanil infusion. If necessary, IV mannitol & moderate hyperventilation used to reduce brain bulk & optimize operating conditions. Normothermia was maintained throughout the case. Crystalloid routinely administered through peripheral IV lines. CVA not used. Indwelling urinary catheters in all pts to monitor urine output. If judged necessary, NMB was reversed at procedure end. | | |
| Postop interventions & imaging | In case of GA extubation in OR. 4 hrs observation in PACU, then DSU transfer for 4-6 additional hrs. CT scan 2-4 hrs postop to exclude surgical complications. Postop pain or PONV were treated according to a standardized protocol, early intake of fluids & solids. | | |
| Discharge, postop course, FU | Predischarge assessment. Discharge criteria: no hemorrhage/edema on CT, stable neurological status, PONV control, overnight availability of caregiver, analgesia & steroid taper prescription, written instructions for pain control, possible adverse symptoms, & FU. Instructions to present to ED if clinical deterioration occurs. | | |
| (B) Levy et al., 2023^29^ | | | |
| Treated pathologies | Different types of brain tumors. | | |
| Eligibility | Pts 16–85 yrs w/ supratentorial tumors with a maximum diameter of 4 cm, ASA ≤ IV, KPS score ≥ 70, treated w/ craniotomy; surgeries with prospectively determined estimated blood loss of < 300 ml, with a maximum predicted time < 3 hours. Exclusion: BMI > 35 kg/m^2^, higher risk of surgical morbidity (e.g., infratentorial, intraventricular, or skull base and pituitary tumors), limited home support or requiring CSF diversion. | | |
| Preop | Admission the evening before surgery, preop clearance and contrast-enhanced MRI. | | |
| Intraop | NA | | |
| Postop interventions & imaging | 3 hrs observation in PACU. Postop contrast-enhanced MRI. 3 hrs observation in ICU. | | |
| Discharge, postop course, FU | Discharge criteria: cleared by the PT and OT teams, remain afebrile, postop imaging without concerning findings, no concerning examination findings, or incisional issues. Pts were seen in the clinic 2 weeks postop. | | |
| (C) Peláez-Sanchez et al., 2023^40^, Peláez-Sanchez et al., 2025^41^ | | | |
| Treated pathologies | Different types of brain tumors. | | |
| Eligibility | Pts >18 yrs old w/ supratentorial tumors treated w/ stereotactic biopsy for deep-seated tumors or craniotomy tumor resection, lesions < 3 cm in diameter and < 1.5 cm deep, caregiver available, patient’s home w/in the geographic area covered by the HaH unit, ≤30 minutes away from the hospital. Exclusion: already an inpatient, significant cardiorespiratory comorbidity, airway management concerns, uncontrolled seizures and/or poor neurological status, duration of surgery >4 hrs, patient preference, chronic comorbidity not satisfactorily controlled. | | |
| Preop | Assessment by anesthesiologist and physicians from the HaH department to ensure suitability for the outpatient procedure. Admisson to the DSU on the day of surgery. | | |
| Intraop | Total IV anesthesia, standard noninvasive monitors, bispectral index monitor. No routine use of urinary catheter and osmotic diuretic, invasive arterial or central lines, and use of continuous noninvasive monitoring instead. Usual surgical procedure w/ standard operative adjuncts (e.g., intraop fluorescence, neuronavigation). Avoidance of long scalp flaps in craniotomy, administration of local anesthesia at the end of the procedure. | | |
| Postop interventions & imaging | 8 hrs observation and monitoring in DSU; initiating oral intake of liquids and progressively of solids; early mobilization with assistance for walking; monitoring micturition; effectively managing nausea, vomiting, and pain through medication. CT scan 6 hrs postop. | | |
| Discharge, postop course, FU | 3 days monitoring by the HaH unit, providing care similar to inpatient hospitalization w/ telematic follow-up and at-home visits. Discharge criteria: absence of any clinical or radiological complications. | | |
| (D) Gurses et al., 2024^24^ | | | |
| Treated pathologies | Different types of brain tumors. | | |
| Eligibility | Age 16–85 years, ASA ≤ IV, KPS ≥ 70, planned same-day discharge after surgery, expected blood loss < 300 ml and duration < 3 h. Exclusion criteria: BMI > 35 kg/m² unless surgeon-approved, need for CSF diversion, lack of adequate home support, surgeon override based on clinical judgment. | | |
| Preop | Admission evening prior to surgery for clearance and MRI, preoperative education by surgical and presurgical staff (ERAS expectations, discharge plan). | | |
| Intraop | NA | | |
| Postop interventions & imaging | 3h of PACU observation, 3h of Neuro-ICU observation (total minimum 6h); early oral intake progression, mobilization, monitoring of voiding, nausea/pain control; mandatory postoperative neuroimaging before discharge. | | |
| Discharge, postop course, FU | Discharge criteria: hemodynamic stability, clean wound, controlled pain, normal imaging, afebrile, independent eating/walking, PT/OT clearance  Decision by attending neurosurgeon prioritizing patient safety  Follow-up clinic visit scheduled 2 weeks post-discharge | | |
| *Early Discharge Protocols* | |  | |
| (A) Sughrue et al., 2015^50^ | | | |
| Treated pathologies | Different types of brain tumors. | | |
| Eligibility | Pts w/ brain tumors treated with elective craniotomy. Exclusion criteria: cranial trauma, hydrocephalus, endoscopic approaches, or stereotactic needle procedures. | | |
| Preop | Antibiotic prophylaxis. | | |
| Intraop | No information about anesthesia. Site-appropriate keyhole craniotomies (2-cm diameter), minimization of soft-tissue manipulation, brain exposure, & brain manipulation as much as possible, no intraop mannitol. | | |
| Postop interventions & imaging | No wound drains & lumbar drains only in rare instances, OR extubation when appropriate, transitional care preferred to ICU. Dexamethasone postop for ≥1 day & stopped in pts w/ extraaxial tumors. In intraaxial tumor pts, steroids tapering off in 3–7 days, depending on length of preop dexamethasone use. Postop pain: avoidance of IV narcotics if not appropriate. Postop imaging: on POD 1 for malignant or intraaxial brain tumor. Low-grade or extraaxial tumors: 6 wks postop unless necessary. | | |
| Discharge, postop course, FU | Early discharge criteria: pt is able to ambulate safely or is able to be adequately cared for at home by family (if they were nonambulatory before surgery). Ability to eat enough food to meet nutritional needs w/o an aspiration risk. Pain well controlled on oral medication. No need for IV medications. Pt awake, alert & has stable neurological exam for ≥6 hrs since awakening from anesthesia. | | |
| (B) Ma et al., 2016^33^ | | | |
| Treated pathologies | Different types of brain tumors. | | |
| Eligibility | Pts w/ intraparenchymal brain tumors treated w/ AC or endoscopic minicraniotomy. Exclusion criteria: emergency op. | | |
| Preop | Outpatients visit w/ neurosurgeon, neurooncology clinical nurse specialist, PT & OT. Pt education on perioperative course & possible complication. Home situation exploration by PT & OT. Local social services & home care team contact details. Preop assessment clinic, at either the same or subsequent visit: routine biochemical & hematological blood tests, ECG & chest x-ray, if indicated. Anesthesia consult if deemed necessary. Early admission day of surgery or night before. Informed consent. CT or MRI for neuronavigation. Final anesthetic review on the day of surgery. | | |
| Intraop | Antibiotic prophylaxis (flucloxacillin & gentamicin IV on induction; ceftriaxone in replacement of flucloxacillin for pts w/ penicillin allergy as per hospital protocol).  AC 🡪 no routine arterial line, noninvasive monitoring, injection w/ bupivacaine-adrenaline of the operative site before surgery. During the opening & closing of the craniotomy, short-acting sedative agents, such as propofol, midazolam, & fentanyl, were titrated to pt comfort.  GA 🡪 arterial line for invasive BP monitoring & intraop gas analysis, dual IV access, ECG monitoring & insertion of bladder catheter. Infiltration w/ bupivacaine-adrenaline of the wound site before surgery. Standard GA. | | |
| Postop interventions & imaging | Transfer to ward, assessment by neurosurgeon, postop MRI or CT (if MRI contraindications) w/in 24 hrs, no AED unless previous Sz. | | |
| Discharge, postop course, FU | Discharge criteria: pt is ambulatory, good pain control, no complications postop imaging, discharge agreed by medical team & pt. Information on postop course. Instruction on complications & possible return to hospital. | | |
| (C) Neville et al., 2020^38^ | | | |
| Treated pathologies | Different types of brain tumors. | | |
| Eligibility | Pts treated w/ brain tumor resection. Exclusion: surgery for hydrocephalus, hematoma, CSF leakage, surgical site infection, cranial reconstruction, endoscopic approach, and stereotactic biopsy. | | |
| Preop | Preop workup w/ basic laboratory tests, chest X-Rays, EKG, preop evaluation by general practitioner and anaesthesiologist. Fasting 8 hrs preop, antithrombotic prophylaxis w/ graduated compression stockings and intermittent pneumatic compression. | | |
| Intraop | Type of anaesthesia, amount of fluid, and intraop management of neuromuscular blockade decided by the attending anaesthesiologist. Conventional total IV anaesthesia was used for GA in pts who did not underwent awake craniotomy. A single dose of prophylactic antibiotics (second generation cephalosporin) 30 min before skin incision and scalp infiltration with 10 ml of 2% lidocaine. Smallest possible craniotomy that allowed a safe brain tumour removal. Perioperative brain mapping with navigated transcranial magnetic stimulation, neuronavigation, and intraop US were used when necessary. Extubated in the OR whenever possible, pts were not extubated in the OR if there were brain oedema leading to transcalvarian herniation after tumour removal, unexpected need to expand the craniotomy, or massive blood transfusion. | | |
| Postop interventions & imaging | Transferred directly to the postop ICU. Postop dexamethasone and phenytoin doses maintained as the preop schedule and weaning managed case by case. Oral feeding reintroduced 6 hours after surgery for patients without dysphagia and with stable neurologic exam. Arterial lines, central venous catheters, and Foley bladder catheter usually removed early the following morning (POD 1). Discharge from ICU was left to the discretion of the critical care staff physicians. Blood tests and CT scan done within 24 hrs postop, repeated as needed. | | |
| Discharge, postop course, FU | Discharge criteria: oral feeding without aspiration risk, pain control with oral medications, no need for IV medications, stable neurological examination, CT scan ruling out early surgical complications, caregiver available and pt agree with discharge | | |
| *Avoidance of Routine ICU Admission Protocols* | | | |
| (A) Ter Laan et al., 2020^51^ | | | |
| Treated pathologies | Different types of brain tumors. | | |
| Eligibility | Pts >18 yrs w/ supratentorial brain tumor treated w/ elective craniotomy. Exclusion criteria: emergency, pediatric, or vascular cases; endoscopic procedures; high expected blood loss; expected >6-hr duration of surgery; cardiopulmonary comorbidity; poor functional status. | | |
| Preop | NA | | |
| Intraop | NA | | |
| Postop interventions & imaging | PACU observation for 1 hr, then transfer to ward. Ward transfer criteria: Aldrete score ≥9, ≥30 mins after last IV opioid & pain under control, adequate urinary production (0.5–1 ml/hr) w/ an indwelling catheter or postop urinary retention ruled out w/ US, temperature ≥36°C, PONV treated & controlled, appropriate analgesic, antiemetic & fluid regimens, no signs of postop bleeding, baseline Modified Early Warning Score measurement recorded. Ward monitoring: routine monitoring of pupillary function, LOC (GCS) & global motor function checked every 15 mins, assessment by an attending neurosurgeon or trainee w/in 1 hr postop, when transferred to the ward monitoring of HR & O2 saturation continued until 6 hrs postop, while LOC & pupils are checked every hr. Alarms set at sO2 <96%, HR <50 or >90 per min, SBP <90 or >160 mm Hg. | | |
| Discharge, postop course, FU | NA | | |
| (B) Hoffman et al., 2023^25^ | | | |
| Treated pathologies | Different types of brain tumors. | | |
| Eligibility | Pts treated w/ supratentorial craniotomy for resection of primary or metastatic tumor, a total case length < 5 hours, estimated blood loss < 500 ml, routine extubation in the operating room without complication, absence of cardiac dysrhythmia intraop or in the PACU, maintenance of a postop systolic blood pressure < 150 mmHg > 1 hr without intravenous antihypertensive medications, at or near their neurological baseline without new postoperative deficits at the time of discharge from the PACU, not at their neurological baseline if permitted by their attending surgeon following neurological evaluation, transient postop deficits that demonstrated rapid improvement. Exclusion: difficult airway as determined by anesthesia service per Anesthesia Airway Placement Note, BMI > 40 or BMI > 30 with a history of obstructive sleep apnea, requiring continuous iv drips, need for nonalbumin blood product transfusion, presence of an intracranial drain (except for a subgaleal drain), an intra- or postop seizure, coagulopathy | | |
| Preop | NA | | |
| Intraop | NA | | |
| Postop interventions & imaging | 4-hour monitoring period in the PACU w/ regular neurological examinations and vital sign checks every 15 minutes for the 1st hour, every 30 minutes for the next 2 hrs, then every hour until transfer to the floor. Samples for laboratory work drawn on patient arrival to monitor hematological stability; the patient’s arterial line is maintained while in the PACU. A neurosurgeon or advance practice provider from the neurosurgery service examines the patient within 30 minutes of arrival and documents the examination by the end of the 4-hour observation period. Multidisciplinary determination after 4 hrs about the disposition of the patient to confirm their continued appropriateness for pathway enrollment and discharge to the floor. An escalation protocol was defined to outline the criteria (incl. intra- or postop Sz, focal postop neurological change or new-onset agitation, cardiopulmonary instability, new cardiac dysrhythmia) by which pathway patients should be transferred to the ICU. | | |
| Discharge, postop course, FU | NA | | |
| (C) Perez-Vega et al., 2023^42^ | | | |
| Treated pathologies | Different types of brain tumors. | | |
| Eligibility | Pts w/ no immediate postoperative complications (intracranial hemorrhage, seizure episode), no intracranial pressure monitor or external ventricular drain placement, no need of volume resuscitation or vasopressors, no need for invasive ventilation, no signs of clinical deterioration. Exclusion: different procedures other than craniotomy for brain tumor (i.e., thrombectomy, aneurysm coiling, cranioplasty, and ventriculoperitoneal shunt). | | |
| Preop | NA | | |
| Intraop | NA | | |
| Postop interventions & imaging | 2 hrs observation on the fifth floor PACU and evaluation by anesthesia, neurocritical care, neurosurgery, and nursing teams for suitability to transfer to NPCU. The checklist-criteria included: no immediate postoperative complications (intracranial hemorrhage, seizure episode), no intracranial pressure monitor or external ventricular drain placement, no need of volume resuscitation or vasopressors, no need for invasive ventilation, and no signs of clinical deterioration. Pt was transferred to NSICU if any of these criteria were unmet.  In the NPCU, the neurocritical care team oversaw rounding and treatment plans; hourly neurological checks during the first 12 hours were performed, then every 4 hours thereafter (routine floor evaluations). If the pt remained clinically stable or progressing (eg, able to tolerate oral administration, or get out of bed with physical therapy) care would be handed off to the neurosurgical floor team. | | |
| Discharge, postop course, FU | NA | | |
| (D) Wagner aet al., 2025^56^ |  | | |
| Treated pathologies | Brain tumors and vascular lesions | | |
| Eligibility | Inclusion criteria: consecutive adult patients (≥17 years) undergoing elective craniotomy, procedures performed from 2013 onward once full digital records were available. Exclusion criteria: NA | | |
| Preop | Preoperative anesthesiology evaluation to identify high-risk patients (ASA ≥4) for planned ICU admission, multidisciplinary consensus (anesthesiology + neurosurgery) on postoperative disposition at least 24h prior to surgery | | |
| Intraop | Intraoperative adverse events triggered immediate joint neurosurgeon-anesthesiologist reassessment to determine direct ICU transfer | | |
| Postop interventions & imaging | Standard postoperative admission to an 8-bed PACU with invasive BP monitoring, ventilator support capability, and neuroanesthesia-trained staff; Monitoring: neurological status, pain, ICP monitoring in patients with EVD, routine labs on POD1 (CBC, coagulation); CT performed on demand after neurosurgical consultation (semi-closed model) | | |
| Discharge, postop course, FU | Morning multidisciplinary PACU rounds on POD1 to determine step-down: regular ward vs IMC vs ICU; PACU also functioned as a preparation unit for revision surgery | | |
| *Awake Craniotomy Protocols* | | | |
| Chen et al., 2021^15^ | | | |
| Treated pathologies | Different types of brain tumors and vascular pathologies. | | |
| Eligibility | Pts treated w/ elective AC. Exclusion: NA | | |
| Preop | Extensive periop counseling, abstination from alcohol and smoking > 2 weeks, antimicrobial prophylaxis, shortened fluid fasts, consumption of oral carbohydrates 2 hrs preop, practice wearing diapers 24 hrs preop, use of 5% compound lidocaine cream (CLC) to relieve the pain of nerve blocks and vascular punctures, adjustment of the room temperature to 25 °C before the pt enters OR. | | |
| Intraop | Monitoring w/ ECG, noninvasive BP measurements, body temperature, end-tidal carbon dioxide, and invasive BP, use of the bispectral index monitoring system to guide requirement for intraop anesthetics; hourly blood gas analysis to assess oxygen delivery to tissue and oxygen consumption of tissue, ultrasound-guided scalp nerve block; continuous infusion of propofol, dexmedetomidine, and sufentanil to maintain the bispectral index value between 60 and 80; equipment for emergency airway control (incl. intubation under direct vision, blind nasal intubation, fiberoptic-assisted intubation, and/or laryngeal mask airway insertion) available throughout the procedure; hemodynamics guided intraop goal-directed fluid therapy; palonosetron 0.15 mg and dexamethasone 10 mg before anesthesia induction; inflatable heated blanket and fluid-heating device; use of diapers. | | |
| Postop interventions & imaging | Encouragement of active or passive exercise from POD 1. | | |
| Discharge, postop course, FU | Discharge standards: absence of dizziness, headache, nausea, vomiting, fever, and other symptoms; well-healed incision; imaging findings showing no intracranial space-occupying hemorrhage or edema; no obvious abnormalities found in the laboratory test results. | | |
| Azghadi et al., 2025^5^ |  | | |
| Treated pathologies | NA | | |
| Eligibility | Pts treated w/ elective AC. Exclusion: NA | | |
| Preop | Pt and family counselling and education 🡪 routine consultation for AC in surgeon’s office, preop anesthesia clinic and preoperative unit: pts were explained the risk and benefit of the AC, the operative staff including surgical staff, anesthesia team, nursing and neurology/neuro monitoring team introduce themselves to the patient to establish connection and trust; Preop evaluation 🡪 pt’s functional capacity, mental status and language capability were assessed prior to the surgery; Preemptive analgesia 🡪 acetaminophen 975 mg p.o. administered at preoperative care unit except for those with allergy or liver dysfunction; Infection prevention and control 🡪 preop MRSA screening, surgical prophylactic antibiotic administration within 1 h prior to skin incision (weight based cefazolin IV if MRSA negative and vancomycin IV if MRSA positive, alternative for those with allergies) | | |
| Intraop | Local anesthetic 🡪 SNB before pinning and subcutaneous local mixture of lidocaine/ bupivacaine/ sodium bicarbonate administered at the incision; Normothermia; Goal-directed fluid therapy; Non-opioid analgesia 🡪 intraop SNB and local incision anesthesia, dexmedetomidine drip intraoperatively | | |
| Postop interventions & imaging | Post analgesia 🡪 multimodal opiod-sparing analgesia; PONV prophylaxis and treatment; Delirium precautions; Early mobilization and  Ambulation; Glycemic control | | |
| Discharge, postop course, FU | Arrange Safe discharge home or to rehabilitation center; Timely follow-up with outpatient clinic visit; Two weeks and three month follow up in surgeon’s clinic | | |
| *Process Improvement Protocols* | | | |
| McLaughlin et al., 2014^34^; McLaughlin et al., 2014^35^ | | | |
| Treated pathologies | Trigeminal neurovascular conflict. | | |
| Eligibility | Pts w/ trigeminal neurovascular conflict. No exclusion criteria reported. | | |
| Preop | Pt education, description of expected postop course, w/ duration of stay in each location; discussion of expected goals for hydration/nutrition, elimination, mobilization, management of postop pain, PONV; discussion of the expected postop LOS (1–2 days) depending on service lines. | | |
| Intraop | Multidisciplinary efforts for parallel pt preparation, introduction of a comprehensive timeout, analysis of OR team’s performance; technical improvements. Simplification of intraop monitoring to decrease cost: BAEPs monitoring & facial nerve electromyography not standard for proficient surgeons. | | |
| Postop interventions & imaging | Pts w/ age ≤65 yrs, w/ no cardiorespiratory comorbidities, & w/ intact neurological exam in the recovery room transported directly to the regular ward. Identification of pts ready to be discharged during afternoon rounds & initiation of discharge preparation, revision of postop order sets, standardization of elements of care. | | |
| Discharge, postop course, FU | Standardization of discharge criteria & standardization of discharge instruction sheets. Steroid taper, suture removal & FU appointment; reviewing each discharge at the daily morning multidisciplinary rounds. | | |
| *Early Mobilization Protocols* | | | |
| Chen et al., 2025^16^ | | | |
| Treated pathologies | Glioma. | | |
| Eligibility | Inclusion criteria: histopathologically confirmed glioma (CT/MRI + pathology), age ≥ 18 years, no prior surgical treatment. Exclusion criteria: other malignant tumors or distant metastases, preoperative limb muscle strength <grade 4 (MRC scale), preexisting neurological deficits (e.g., aphasia, neglect, ataxia, visual deficits), history of cardiac, cerebrovascular, or hepatic disease, mental illness or severe psychological disorders. | | |
| Preop | Health education materials and communication support groups prepared for early mobilization awareness | | |
| Intraop | Standardized multimodal analgesia approach including preemptive analgesia and anesthesia strategies to address central sensitization | | |
| Postop interventions & imaging | Head elevation for venous drainage, high-protein/high-vitamin diet as tolerated; Pain: Daily assessments, multimodal analgesia, intervention for pain ≥2; Mobilization Protocol (Day 0–7): Progressive, nurse-supervised activity escalation based on muscle strength (MRC scale), starting with bed exercises → sitting → standing → assisted walking → independent ambulation (goal ≥2h/day by Day 7); Infrastructure Support: Handrails, walking aids, educational videos, motivational signage, communication groups | | |
| Discharge, postop course, FU | NA | | |
| Zarrin et al., 2025^65^ |  | | |
| Treated pathologies | Brain tumors | | |
| Eligibility | Inclusion criteria: adults ≥18 years, elective craniotomy for tumor resection between 2017–2024. Exclusion criteria: same-day discharge, stereotactic radiosurgery lesions, primary spinal pathology, normal pressure hydrocephalus, IV analgesic infusion >24h post-op, multiple resections, in-hospital mortality | | |
| Preop | NA | | |
| Intraop | Standard institutional cranial resection anesthesia and surgical protocols (not modified by NERVS) | | |
| Postop interventions & imaging | Nurse-driven standardized ERAS-like mobilization pathway:   - POD0: sit upright within 4h of surgery for 5 min, repeat every 2h 🡪 walk 5 ft to chair - POD1+: sit in chair ≥3× daily 🡪 walk 50 ft with RN 🡪 hallway ambulation with reduced assistance - Pain: baseline assessment, swallow evaluation before POD1, non-pharmacologic pain strategies prioritized - Education: medication cards, discharge care teaching, follow-up planning - Failure protocol: if goal not achieved 🡪 retry in 6–8h; persistent failure 🡪 trigger formal PT/OT discharge pathway | | |
| Discharge, postop course, FU | NA | | |
| *Comprehensive ERAS Protocols* | | | |
| Wang et al., 2018^57^; Liu et al., 2019^30^; Liu et al., 2020^32^; Qu et al., 2020^44^; Wang et al., 2022^58^; Wang et al., 2022^59^; Yakar et al., 2023^61^ | | | |
| Treated pathologies | Different types of intracranial pathologies. | | |
| Eligibility | Pts 18-65 yrs, medically eligible for elective craniotomy. Exclusion criteria: cranial trauma, pathology requiring emergency op, preop disturbance of consciousness, confounding condition (e.g., pregnancy), disease that could impact postop recovery (e.g., paralysis, spinal deformity, autoimmune diseases, myocard infarct, liver & renal malfunction, or severe mental illness. | | |
| Preop | Functional status evaluation (KPS); smoking & alcohol abstinence; anxiety & depression evaluation (HADS); evaluation of prophylactic antithrombotic therapy (VTE Caprini Risk Assessment & Autar DVT Risk Assessment Scale); PONV risk score assessment; preop intestinal intervention (if long history of constipation or ≥2 w/o defecation); nutritional assessment (NRS2002) skin preparation (hair washing w/ chlorhexidine, shaving 1.5-2 cm beyond incision); antimicrobial prophylaxsis (cefazolin 1 hr prior to skin incision, 2^nd^ dose if surgery >4 hrs); preop enteral nutrition, oral carbohydrate loading (fasting solid fluid for 6 hrs & oral intake of maltodextrin fructose solution morning of op day) & periop oral immune nutrition. | | |
| Intraop | Scalp blocks (add dose if operational time >3 hrs), IV-inhalation combined anesthesia, hypothermia avoidance, GDFR strategy (noninvasive cardiac output monitoring to keep vol status & hemodynamic stability). Minimally invasive craniotomies & endoscopic skull base approaches, absorbable skin suture (intradermal), no drainage unless specific circumstances, if places remove w/in 48 hrs. | | |
| Postop interventions & imaging | Early removal of urinary drain (w/in 6 hrs); PONV management/prevention; postop pain control w/ nonopioid and opioid analgesia. CT scan on POD 1, MRI w/in 3 days postop. | | |
| Discharge, postop course, FU | Early mobilization (bed exercise 6 hrs after awaking, early ambulation 24 hrs postop. Rapid de-escalation of fluids. Nutritional assessment (NRS2002), anxiety & depression evaluation (HADS). Discharge criteria: adequate pain management w/ oral analgesia, adequate intake of solid food w/o need for IV fluids, no fever, independent mobility, & safe disposition home. FU: outpatient visit 2 wks & 4 mos after discharge. | | |
| Wu et al., 2021^60^ | | | |
| Treated pathologies | Different types of brain tumors. | | |
| Eligibility | Pts >18 yrs w/ a clear diagnosis of supratentorial tumor requiring surgical operation, diagnosed by brain CT and MRI, of both genders, ASA I-II, able to cooperate and sign the informed consent voluntarily. Exclusion: refusal to participate or withdrawal halfway, poor compliance, encountering other stress events that may affect the results of the study, incomplete case data | | |
| Preop | Preop assessment: nutritional risk screening w/ VAS and the facial expression scale to evaluate the pain, Barthel index score, DVT risk assessment. ERAS education: information on the concept of ERAS and explanation of the operation methods and complications through PPT.  Preop airway preparation: quit smoking 4 weeks preop. Training methods (e.g. climbing stairs and blowing balloons) were encouraged to increase vital capacity. Pts were taught how to breathe deeply and cough effectively. Family members were taught how to assist them to turn over and tap the back effectively.  Prevention of DVT: pts > 60 years or admitted to the hospital with a high-risk VTE score were informed about the VTE risk and preventive measures (e.g. family members and pts were thaught how to perform passive exercise training, ankle pump exercises, prepare elastic stockings, and were guided on correct wearing methods preop)  Preop gastrointestinal management: fasting 6 hrs preop, pts without a history of diabetes orally took 400 ml of carbohydrate solution 3 hrs preop. Use of cathartic 1 day preop and glycerine enema if necessary;  Preop medication: history of allergies was assessed and skin test was performed. Antibacterial drugs were prepared for IV prophylactic application on the day of the operation  Skin preparation: shampoo containing “4% chlorhexidine” was used to clean the head 3 days preop.The marginal hair was trimmed on the day of surgery. The 3M surgical skin preparation kit was used for partial haircuts (2–3 cm wide along the incision). The rest of the hair was sterilized and long hair was given a split braid. | | |
| Intraop | NA | | |
| Postop interventions & imaging | Postop monitoring: The NICU care was performed on the first day after surgery.  Close observation of the vital signs and complications  4 hrs after awake extubation, pts were orally or nasally fed with clear liquid nutritional supplements, 30 to 50 ml/time, once every 1 to 2 h.  Pt was sent back to the neursurgery ward if CT showed no hemorrhage, ischemia, edema, and other intracranial abnormalities 24 hrs postop  Postop gastrointestinal management: routine use of antiemetics 3 days postop. Early nutritional support, 100 to 200 ml/time added every 2 to 3 h from 24 hrs postop, gradually transferred to semi liquid according to the tolerance. Ordinary food was given 72 hrs postop for those without eating difficulties  Prevention of postoperative deep vein thrombosis: High risk VTE patients stopped using hemostatic drugs 2 days postop, and use graded pressure socks and intermittent pneumatic pump until discharge. Routine use of anticoagulants when pts had obvious thromboembolic symptoms  Postop tube care: The negative pressure drainage ball was removed within 48 hrs. Pts were introduced to start bladder function training 12 hrs postop.  The pts should urinate every 3 to 4 h within 24 hrs postop. The central venous catheter was removed within 72 hrs postop  Postop pain management: VAS and facial expression scale to score postop pain and reevaluated according to the doctor’s analgesic instructions; early postop getting out of bed activities. | | |
| Discharge, postop course, FU | PQRS scales are completed by telephone follow-up at the end of the operation, on POD 3, 1 week postop and 1-3 months postop. | | |
| Elayat et al., 2021^19^ | | | |
| Treated pathologies | Different types of brain tumors. | | |
| Eligibility | Pts > 18 yrs w/ single supratentorial space-occupying lesion treated w/ elective craniotomy, ASA I-II. Exclusion: moribund patients requiring emergency craniotomies, uncontrolled diabetics, severely cognitively impaired patients unable to follow simple instructions | | |
| Preop | Family education, complex-carbohydrate drink, flupiritine. | | |
| Intraop | Scalp blocks, limited opioids, rigorous fluid and temperature regulation. | | |
| Postop interventions & imaging | Flupiritine, early mobilization, removal of catheters, and initiation of feeds. | | |
| Discharge, postop course, FU | Discharge criteria: adequate pain control, afebrile state, cardiopulmonary stability, being able to sit out of bed. | | |
| Feng et al., 2021^20^ | | | |
| Treated pathologies | Different types of brain tumors. | | |
| Eligibility | Pts 18–65 yrs w/ preop diagnosis brain tumor, no obvious neurological and cognitive impairment preop, no serious concomitant diseases that affect the prognosis. Exclusion: NA | | |
| Preop | Preop evaluation of vomiting risk and application of antiemetics. 200 ml of carbohydrates was taken orally 2 hrs preop. Assessment of preoperative thrombosis risk and prevention of postop thrombosis. | | |
| Intraop | Scalp nerve block and local infiltration anesthesia with ropivacaine. Indwelling wound drainage tube intraop was avoided. | | |
| Postop interventions & imaging | Oral feeding resumed as early as possible postop. Urinary catheter removed as soon as possible postop. Early ambulation training with the help of nurses postop. | | |
| Discharge, postop course, FU | 1 month follow-up period. | | |
| Abhinav et al., 2023^1^ | | | |
| Treated pathologies | Different types of brain tumors. | | |
| Eligibility | Pts 18-65 yrs w/ one intracranial lesion, medically qualified for an elective craniotomy, who can interact effectively with medical professionals, with adequate research adherence. Exclusion: conditions other than brain tumors, awake cranial surgery, serious spinal cord shock, additional injuries brought on by preceding cardiac arrest along with severe limb, thoracic, or abdominal injuries, infection or swelling near the surgical site, severe comorbidities, advanced heart disease, impaired functioning of the liver and kidneys, mental illness, gave birth within 6 months prior to this study or breastfeeding. | | |
| Preop | Pt education, nutritional support, prehabilitation. | | |
| Intraop | Standardized anesthesia protocols, minimally invasive surgical technique. | | |
| Postop interventions & imaging | Early mobilization, multimodal pain management, nutrition. | | |
| Discharge, postop course, FU | 4 month follow-up period. | | |
| Kaewborisutsakul et al., 2023^27^ | | | |
| Treated pathologies | Different types of brain tumors. | | |
| Eligibility | Pts 15+ yrs w/ single brain lesion treated w/ elective craniotomy, managed with the ERAS protocol in the study. Exclusion: profound weakness, dependent status, uncontrolled cardiovascular diseases, severe metabolic diseases, sepsis, history of neuropsychiatric disease, severe cognitive impairment, pregnancy | | |
| Preop | Comprehensive preop evaluation and counseling conducted by neurosurgeon; abstinent from smoking and alcohol consumption ≥ 4 weeks; nutritional assessment and dietary advise; deep breathing exercise; appliance of mouthwash with chlorhexidine solution after admission till discharge; washing hair with chlorhexidine scrub during adimission and POD 2; fasting solid food for 8hrs preop; oral intake clear carbohydrate drink 2-4h preop; acetaminophen 500–1000 mg per oral 2–4h preop. | | |
| Intraop | Scalp block w/ bupivacaine injection for ipsilateral side in unilateral scalp incision or both sides for bicoronal incision; local anesthesia uses 1% Xylocaine with adrenaline 1:200,000 infiltrated along subcutaneous scalp incision; risk assessment with institute's protocol which use simplified risk factor from Apfel's score (female, smoking, history of PONV/motion sickness, intraoperative opioid use); cefazolin administration within 1h prior to scalp incision; hair shaving about 2cm over the incision line; subcutaneous and skin are sutured by absorbable suture. | | |
| Postop interventions & imaging | Water intake w/in 8 hrs after extubation; normal solid diet within 24 hrs after extubation; early removal of urinary catheter within 24 hrs postop; start bed exercises and/or ambulation program within 24hrs postop; ultrasound leg vein for screening DVT during admission; intermittent pneumatic calf compression. CT scan on POD 1. | | |
| Discharge, postop course, FU | NA | | |
| Khan et al., 2023^28^ | | | |
| Treated pathologies | Different types of brain tumors. | | |
| Eligibility | Pts >16 yrs w/ nonvascular and non-infectious supratentorial lesions treated w/ elective craniotomy, KPS ≥ 80, absence of significant coagulopathies, adequate home support, low medical comorbidity burden (mCCI ≤ 6). Exclusion: suprasellar lesions, unexpected intraoperative events, any skull base violation raising concern for CSF leakage, any requirement for blood product transfusion, persistent antihypertensive or pressor requirement postop, failure to return to expected neurological baseline within 8 hrs of surgery, recommendation against home discharge by PT or OT, presence of any unexpected findings on postop imaging | | |
| Preop | Perioperative counseling for pts and caregivers, received handouts detailing the expected perioperative course. Nursing staff, neurosurgical residents, neurosurgical advanced practice providers, and physical/occupational therapists were all prenotified of patients enrolled in the pathway to better facilitate care coordination. Nursing staff received an in-training session describing the pathway and its goals prior to implementation. | | |
| Intraop | NA | | |
| Postop interventions & imaging | Postop to the neurological ICU postoperatively. Hourly neurological examination checks overnight. Removal of urinary catheters and mobilization was attempted within 8 hrs postop.  POD 1: early PT and OT sessions with ambulatory mobilization. Unexpected findings on postop imaging (incl. any significant hemorrhage, infarct, or mass) resulted in disqualification from the pathway effect.  POD 2: early PT and OT sessions, removal of surgical subgaleal drains, discharge home. | | |
| Discharge, postop course, FU | Postdischarge day 1 (i.e., POD3) ; telehealth visits with a neurosurgical nurse practitioner or physician assistant for postsurgical assessment. Directions to return to the ED for further evaluation if there were any concerns. | | |
| Sivakumar et al., 2023^48^ | | | |
| Treated pathologies | Different types of brain tumors. | | |
| Eligibility | NA  NA | | |
| Preop | Patient counseling and optimization (e.g., smoking cessation, alcohol abstinence, nutritional optimization) | | |
| Intraop | Short-acting anesthetics, multimodal analgesia (opiate-sparing, local/regional block), PONV prevention, hypothermia prevention, prophylactic antibiotics, glycemic control | | |
| Postop interventions & imaging | Multimodal analgesia (opiate-sparing), early mobility, DVT prevention, glycemic control, concise discharge instructions, continuation of nutritional optimization | | |
| Discharge, postop course, FU | Telephone calls on postdischarge day 1 to reiterate postoperative care expectations. Ultra-early virtual or in-person clinic visit, between postoperative days 4 and 7, to ensure protocol adherence and answer patient questions. | | |
| Zaed et al., 2023^64^ | | | |
| Treated pathologies | Glioma. | | |
| Eligibility | Pts >75 yrs w/ single primary intracranial lesion suspected of high-grade glioma (HGG), medically and surgically eligible for surgical resection. Exclusion: requiring immediate care (incl. pathologies requiring emergency op or those with preop disturbances of consciousness), diseases that could impact postop recovery, unable to follow the instructions of ERAS elements | | |
| Preop | Exhaustive explanation of benefits of abstaining from alcohol and smoking, and of early ambulation and discharge from ICU; flupiritine maleate (100 mg) 2 hrs preop; maltodextrin 100 mL 2 hrs preop; DM measured and managed. | | |
| Intraop | Scalp blocks w/ bupivacaine 0.25% given after the inductions; ensurance with warm IV fluids, forced air warmers, monitoring of body tempterature; goal-directed approach; as minimally invasive as allowed; blake drainage tube only used in special circumstance. | | |
| Postop interventions & imaging | Avoidance of opiods; early mobilization; foley’s catheter removed the first day, after mobilization; oral sips encouraged immediately 2 hrs postop; DVT pumps immediately postop, chemical prophylaxis considered only in plegic patients. | | |
| Discharge, postop course, FU | Discharge criteria: reasonable VAS score with oral antalgetics, no signs of clinical postop infection, intake of solid food, independent mobility, safe disposition at home. | | |
| Abul et al., 2024^2^ | | | |
| Treated pathologies | Different types of intracranial pathologies. | | |
| Eligibility | Pts >18 yrs treated w/ elective cranial surgery, medically fit, required no input from an allied surgical specialty. Exclusion: cochlear nerve dysfunction and underwent joint ENT-neurosurgical intervention. | | |
| Preop | Counselling and education for ERACraS journey and post-operative recovery; smoking and alcohol cessation counselling 4 weeks preop; nutritional status screening with MUST scoring, dietetic input if malnourished; avoiding IV fluids preop, allowing clear fluids up to 2 hrs preop. | | |
| Intraop | Absorbable sutures; reducing time under GA - theatre efficiency, early extubation in theatre if no airway complications; reducing operative time; avoid hypothermia; local anesthesia and nerve blocks; avoiding wound drains; avoiding urethral catheters; single dose of prophylactic antibiotics within 60 min of incision (local guidelines). | | |
| Postop interventions & imaging | Prompt discharge when safe; service contact details on discharge; early enteral feeding - aim for full diet at D0 postop; reducing adverse effects of opiate prescription (laxatives, anti-emetics); multimodal analgesia prescribed by the anaesthetic team after post-operative assessment in recovery; avoiding opioids; early catheter removal when used; prompt critical care admission when necessary; prompt radiological investigation when complication(s) suspected; early mobilization, early de-escalation of IV fluids at D0 postop; early return to enteral feeding at D0 postop. Smoking and alcohol cessation counselling continued for 4 weeks postop. | | |
| Discharge, postop course, FU | Discharge criteria: baseline GCS, no new neurological deficit; tolerating full diet; tolerating oral analgetics and antiemetics; no IV medication necessary; no active medical issues; caregiver available at home; pt and caregiver comfortable with discharge. | | |
| Staartjes et al., 2024^49^ | | | |
| Treated pathologies | Chronic subdural hematoma or hygroma | | |
| Eligibility | Pts ≥ 18 yrs w/ subdural chronic hematoma or hygroma treated w/ burr hole surgery. Exclusion: procedures done for subdural empyema/abscesses or primarily planned as craniotomies | | |
| Preop | In cases of anemia: for elective procedures according to institutional patient blood management protocol. Preop testing of thrombocyte count, plasmatic coagulation, and fibrinogen. Substitution if necessary. In case of antiaggregation or anticoagulation, elective cases put on hold and emergency cases are carried out under reversal. Cranial imaging ≤ 2 weeks old. Infection prophylaxis w/ cefuroxime or clindamycin. | | |
| Intraop | Prevention of fluid disbalance and blood transfusion. Prevention of hypothermia. Extended intraop monitoring. GA in most cases; in select cases of multiple comorbidities in adequate pts without aphasia undergoing unilateral surgery, local anesthesia can be used. Frontal and parietal burr hole location. Subperiosteal drainage site; no drainage is left in place if the brain immediately approximates the calvarium intraoperatively. In patients without hair over the frontal burr hole, use of a burr hole cover considered. | | |
| Postop interventions & imaging | 6 hrs – 1 night observation on neurosurgical intermediate care unit, then transfer to neurosurgical ward. Thromboprophylaxis started 6 hrs postop; postop rest with the upper body 30° elevated; allowed to sit up and mobilize 6 hrs postop; drain does not need to be clamped, when sitting up or mobilizing; the drainage bag generally held at heart level, removal after 36–48 hrs in supine position. No imaging up to discharge as long as clinically stable. Physiotherapy and ergotherapy in all cases with focal deficits, cognitive deficits, or gait insecurity; early removal or avoidance of urinary catheters. | | |
| Discharge, postop course, FU | Follow-up outpatient visit and noncontrast CT scan 6 weeks postop. Oral anticoagulants and antiaggregants are started again 1 day after suture/staple removal (done on POD 8). | | |
| Vedicherla et al., 2025^54^ |  | | |
| Treated pathologies | Chronic and subacute subdural hematoma | | |
| Eligibility | Inclusion criteria: adult patients with surgically evacuated cSDH or subacute SDH. Exclusion criteria: conservatively managed SDH, acute SDH treated with craniotomy/craniectomy, subdural empyema | | |
| Preop | Considering loculated vs nonloculated morphologies to determine no. of burr holes required to achieve adequate clearance of subdural hemorrhage | | |
| Intraop | Burr holes preferred over minicraniotomy (MC) 🡪 MC only used as salvage treatment w/ inadequate clearance or hemostasis intraop; 1 burr hole is noninferior to 2 burr holes; use of periop drainage advocated to reduce recurrence; subperiosteal drainage preferred over subdural drainage; op under LA preferred | | |
| Postop interventions & imaging | Drain removal 24 hrs postop; allow pt to sit up on POD 0 & ambulate by POD 1; no routine postop imaging; all recurrent cases referred for consideration of MMA embolization if emergent op not required; pathway-based active review of all POD 1 pts | | |
| Discharge, postop course & FU | NA | | |

AC, awake craniotomy; AED, antiepoleptic drug; BAEPs, brainstem auditory evoked potentials; BP, blood pressure; CVA, central venous access; CSF, cerebrospinal fluid; DSU, day surgery unit; DVT, deep vein thrombosis; ECG, electrocardiogram; FU, follow-up; GA, general anesthesia; GDFR, goal-directed fluid restriction; HADS, Hospital Anxiety and Depression Scale; HaH, Hospital-at-Home; HR, heart rate; hrs, hours; ICU, intensive care unit; intraop, intraoperative; IV, intravenous; KPS, Karnofsky performance score; LA, local anesthesia; LOC, level of consciousness; LOS, length of stay; MRI, magnetic resonance imaging; mos, months; NCCU, neurosurgical stepdown unit; NMB, neuromuscular blockade; NRS2002, nutritional risk screening 2002; op, operation; postop, postoperative; OR, operating room; OT, occupational therapy; PACU, post-anesthesia care unit; POD, postop day; PONV, postoperative nausea and vomiting; preop, preoperative; PT, physical therapy; pts, patients; PQRS, Post-operative Quality Recovery Scale; Sz, seizure; US, ultrasound; VTE, venous thromboembolism; yrs, years; w/, with; w/o, without.

# **REFERENCES**

1.	Abhinav K, Jadhav D, Andar UB, Karmarkar V, Agrawal R, Agrawal A. Management of Post-craniotomy Pain in Elective Cases: A Randomized Controlled Trial. *Cureus*. 15(9):e46189. doi:10.7759/cureus.46189

2.	Abul MH, Sescu D, White MA, et al. Enhanced recovery in cranial surgery (ERACraS) - A single-centre quality improvement study. *Clin Neurol Neurosurg*. 2024;236:108095. doi:10.1016/j.clineuro.2023.108095

3.	Akcil EF, Dilmen OK, Vehid H, Ibısoglu LS, Tunali Y. Which one is more effective for analgesia in infratentorial craniotomy? The scalp block or local anesthetic infiltration. *Clin Neurol Neurosurg*. 2017;154:98-103. doi:10.1016/j.clineuro.2017.01.018

4.	Au K, Bharadwaj S, Venkatraghavan L, Bernstein M. Outpatient brain tumor craniotomy under general anesthesia. *J Neurosurg*. 2016;125(5):1130-1135. doi:10.3171/2015.11.JNS152151

5.	Azghadi A, Sharpe M, Mikofalvy K, et al. Role of modified enhanced recovery after surgery (mERAS) in awake craniotomy performed under monitored anesthesia care (MAC); a single center retrospective study. *BMC Anesthesiol*. 2025;25(1):153. doi:10.1186/s12871-025-02983-6

6.	Bala R, Gehlaut P, Mittal K, Singh I. Ropivacaine instillation through subgaleal drain: A novel approach for acute post-craniotomy pain. *Indian J Anaesth*. 2022;66(7):498-504. doi:10.4103/ija.ija_646_21

7.	Bastola P, Bhagat H, Wig J. Comparative evaluation of propofol, sevoflurane and desflurane for neuroanaesthesia: A prospective randomised study in patients undergoing elective supratentorial craniotomy. *Indian J Anaesth*. 2015;59(5):287-294. doi:10.4103/0019-5049.156868

8.	Beauregard CL, Friedman WA. Routine use of postoperative ICU care for elective craniotomy: a cost-benefit analysis. *Surg Neurol*. 2003;60(6):483-489; dicussion 489. doi:10.1016/s0090-3019(03)00517-2

9.	Bernstein M. Outpatient craniotomy for brain tumor: a pilot feasibility study in 46 patients. *Can J Neurol Sci J Can Sci Neurol*. 2001;28(2):120-124. doi:10.1017/s0317167100052781

10.	Bhagat H, Dash HH, Bithal PK, Chouhan RS, Pandia MP. Planning for early emergence in neurosurgical patients: a randomized prospective trial of low-dose anesthetics. *Anesth Analg*. 2008;107(4):1348-1355. doi:10.1213/ane.0b013e31817f9476

11.	Bojaxhi E, Louie C, ReFaey K, et al. Reduced Pain and Opioid Use in the Early Postoperative Period in Patients Undergoing a Frontotemporal Craniotomy under Regional vs General Anesthesia. *World Neurosurg*. 2021;150:e31-e37. doi:10.1016/j.wneu.2021.02.009

12.	Boulton M, Bernstein M. Outpatient brain tumor surgery: innovation in surgical neurooncology. *J Neurosurg*. 2008;108(4):649-654. doi:10.3171/JNS/2008/108/4/0649

13.	Carella M, Tran G, Bonhomme VL, Franssen C. Influence of Levobupivacaine Regional Scalp Block on Hemodynamic Stability, Intra- and Postoperative Opioid Consumption in Supratentorial Craniotomies: A Randomized Controlled Trial. *Anesth Analg*. 2021;132(2):500-511. doi:10.1213/ANE.0000000000005230

14.	Cata JP, Saager L, Kurz A, Avitsian R. Successful extubation in the operating room after infratentorial craniotomy: the Cleveland Clinic experience. *J Neurosurg Anesthesiol*. 2011;23(1):25-29. doi:10.1097/ANA.0b013e3181eee548

15.	Chen YJ, Nie C, Lu H, et al. Monitored Anesthetic Care Combined with Scalp Nerve Block in Awake Craniotomy: An Effective Attempt at Enhanced Recovery After Neurosurgery. *World Neurosurg*. 2021;154:e509-e519. doi:10.1016/j.wneu.2021.07.069

16.	Chen X, Wan L, Wang B. Early mobilization in postoperative glioma patients real world impact on recovery and long term prognosis. *Sci Rep*. 2025;15(1):18032. doi:10.1038/s41598-025-01871-w

17.	Choi S, Choi YH, Lee HS, et al. Effects of Scalp Nerve Block on the Quality of Recovery after Minicraniotomy for Clipping of Unruptured Intracranial Aneurysms : A Randomized Controlled Trial. *J Korean Neurosurg Soc*. 2023;66(6):652-663. doi:10.3340/jkns.2023.0010

18.	Djian MC, Blanchet B, Pesce F, et al. Comparison of the time to extubation after use of remifentanil or sufentanil in combination with propofol as anesthesia in adults undergoing nonemergency intracranial surgery: a prospective, randomized, double-blind trial. *Clin Ther*. 2006;28(4):560-568. doi:10.1016/j.clinthera.2006.04.001

19.	Elayat A, Jena SS, Nayak S, Sahu RN, Tripathy S. Enhanced recovery after surgery - ERAS in elective craniotomies-a non-randomized controlled trial. *BMC Neurol*. 2021;21(1):127. doi:10.1186/s12883-021-02150-7

20.	Feng S, Xie B, Li Z, et al. Retrospective Study on the Application of Enhanced Recovery After Surgery Measures to Promote Postoperative Rehabilitation in 50 Patients With Brain Tumor Undergoing Craniotomy. *Front Oncol*. 2021;11:755378. doi:10.3389/fonc.2021.755378

21.	Gerlach K, Uhlig T, Hüppe M, et al. Remifentanil-propofol versus sufentanil-propofol anaesthesia for supratentorial craniotomy: a randomized trial. *Eur J Anaesthesiol*. 2003;20(10):813-820.

22.	Goettel N, Chui J, Venkatraghavan L, Tymianski M, Manninen PH. Day surgery craniotomy for unruptured cerebral aneurysms: a single center experience. *J Neurosurg Anesthesiol*. 2014;26(1):60-64. doi:10.1097/ANA.0b013e3182991d8b

23.	Grundy PL, Weidmann C, Bernstein M. Day-case neurosurgery for brain tumours: the early United Kingdom experience. *Br J Neurosurg*. 2008;22(3):360-367. doi:10.1080/02688690801961858

24.	Gurses ME, Khalafallah AM, Gecici NN, et al. Optimizing outpatient neurosurgery: evaluating ambulatory surgery and same-day discharge following intracranial tumor surgery and endoscopic third ventriculostomy. *J Neurosurg*. 2024;141(6):1635-1643. doi:10.3171/2024.4.JNS24504

25.	Hoffman SE, Gupta S, O’Connor M, et al. Reduced time to imaging, length of stay, and hospital charges following implementation of a novel postoperative pathway for craniotomy. *J Neurosurg*. 2023;139(2):373-384. doi:10.3171/2022.12.JNS222123

26.	Kadian S, Gupta P, Agrawal S. Comparative Evaluation of analgesic efficacy of ketamine and magnesium sulfate as adjuvants to bupivacaine for scalp block in supratentorial Craniotomy: A Randomized, Double-Blind clinical study. *J Clin Neurosci Off J Neurosurg Soc Australas*. 2025;137:111347. doi:10.1016/j.jocn.2025.111347

27.	Kaewborisutsakul A, Kitsiripant C, Kaewsridam S, Kaewborisutsakul WK, Churuangsuk C. The influence of enhanced recovery after surgery protocol adherence in patients undergoing elective neuro-oncological craniotomies. *World Neurosurg X*. 2023;19:100196. doi:10.1016/j.wnsx.2023.100196

28.	Khan HA, Hill TC, Suryadevara CM, et al. Development and implementation of an Enhanced Recovery After Cranial Surgery pathway following supratentorial tumor resection at a tertiary care center. *Neurosurg Focus*. 2023;55(6):E4. doi:10.3171/2023.9.FOCUS23552

29.	Levy AS, Merenzon MA, Bhatia S, et al. Same-day discharge after craniotomy for brain tumor resection: enhancing patient selection through a prognostic scoring system. *Neurosurg Focus*. 2023;55(6):E8. doi:10.3171/2023.9.FOCUS23312

30.	Liu B, Liu S, Wang Y, et al. Neurosurgical enhanced recovery after surgery (ERAS) programme for elective craniotomies: are patients satisfied with their experiences? A quantitative and qualitative analysis. *BMJ Open*. 2019;9(11):e028706. doi:10.1136/bmjopen-2018-028706

31.	Liu B, Wang Y, Liu S, et al. A randomized controlled study of preoperative oral carbohydrate loading versus fasting in patients undergoing elective craniotomy. *Clin Nutr Edinb Scotl*. 2019;38(5):2106-2112. doi:10.1016/j.clnu.2018.11.008

32.	Liu B, Liu S, Wang Y, et al. Impact of neurosurgical enhanced recovery after surgery (ERAS) program on health-related quality of life in glioma patients: a secondary analysis of a randomized controlled trial. *J Neurooncol*. 2020;148(3):555-567. doi:10.1007/s11060-020-03548-y

33.	Ma R, Livermore LJ, Plaha P. Fast Track Recovery Program After Endoscopic and Awake Intraparenchymal Brain Tumor Surgery. *World Neurosurg*. 2016;93:246-252. doi:10.1016/j.wneu.2016.06.015

34.	McLaughlin N, Upadhyaya P, Buxey F, Martin NA. Value-based neurosurgery: measuring and reducing the cost of microvascular decompression surgery. *J Neurosurg*. 2014;121(3):700-708. doi:10.3171/2014.5.JNS131996

35.	McLaughlin N, Buxey F, Chaw K, Martin NA. Value-based neurosurgery: the example of microvascular decompression surgery. *J Neurosurg*. 2014;120(2):462-472. doi:10.3171/2013.9.JNS13663

36.	Nassiri F, Li L, Badhiwala JH, et al. Hospital costs associated with inpatient versus outpatient awake craniotomy for resection of brain tumors. *J Clin Neurosci Off J Neurosurg Soc Australas*. 2019;59:162-166. doi:10.1016/j.jocn.2018.10.110

37.	Negm EM, Younus MA, Morsy AA, et al. Opioid-sparing multimodal analgesia for post-craniotomy pain: a randomized, double-blind, placebo-controlled trial. *BMC Anesthesiol*. 2025;25(1):437. doi:10.1186/s12871-025-03306-5

38.	Neville IS, Ureña FM, Quadros DG, et al. Safety and costs analysis of early hospital discharge after brain tumour surgery: a pilot study. *BMC Surg*. 2020;20(1):105. doi:10.1186/s12893-020-00767-y

39.	Nijs K, McQueen S, Chhabra S, Goldmacher J, Venkatraghavan L, Bernstein M. Same-day discharge after craniotomy for tumor resection: a retrospective observational single-center study of 630 patients. *J Neurosurg*. 2024;140(6):1519-1526. doi:10.3171/2023.9.JNS231454

40.	Pelaez-Sanchez CA, Pajaron-Guerrero M, Rodríguez-Caballero A, et al. Enhanced recovery and same-day discharge after brain tumor surgery under general anesthesia: initial experience with Hospital-at-Home-based postoperative follow-up. *Neurosurg Focus*. 2023;55(6):E6. doi:10.3171/2023.9.FOCUS23550

41.	Peláez-Sanchez CA, Pajarón-Guerrero M, Rodriguez-Caballero A, et al. Cost Analysis of Oncological Outpatient Neurosurgery Under General Anesthesia with Hospital-At-Home-Based Postoperative Care. *World Neurosurg*. 2025;193:1002-1007. doi:10.1016/j.wneu.2024.10.093

42.	Perez-Vega C, Sanghavi DK, Moreno Franco P, et al. Safety and Feasibility of a Fast-Track Pathway for Neurosurgical Craniotomy Patients: Bypassing the Intensive Care Unit. *Mayo Clin Proc Innov Qual Outcomes*. 2023;7(6):534-543. doi:10.1016/j.mayocpiqo.2023.09.002

43.	Purzner T, Purzner J, Massicotte EM, Bernstein M. Outpatient brain tumor surgery and spinal decompression: a prospective study of 1003 patients. *Neurosurgery*. 2011;69(1):119-126; discussion 126-127. doi:10.1227/NEU.0b013e318215a270

44.	Qu L, Liu B, Zhang H, et al. Management of Postoperative Pain after Elective Craniotomy: A Prospective Randomized Controlled Trial of a Neurosurgical Enhanced Recovery after Surgery (ERAS) Program. *Int J Med Sci*. 2020;17(11):1541-1549. doi:10.7150/ijms.46403

45.	Rajan S, Hutcherson MT, Sessler DI, et al. The Effects of Dexmedetomidine and Remifentanil on Hemodynamic Stability and Analgesic Requirement After Craniotomy: A Randomized Controlled Trial. *J Neurosurg Anesthesiol*. 2016;28(4):282-290. doi:10.1097/ANA.0000000000000221

46.	Rigamonti A, Garavaglia MM, Ma K, et al. Effect of bilateral scalp nerve blocks on postoperative pain and discharge times in patients undergoing supratentorial craniotomy and general anesthesia: a randomized-controlled trial. *Can J Anaesth J Can Anesth*. 2020;67(4):452-461. doi:10.1007/s12630-019-01558-7

47.	Sarmento SA, Rabelo NN, Figueiredo EG. Minimally Invasive Technique (Nummular Craniotomy) for Mesial Temporal Lobe Epilepsy: A Comparison of 2 Approaches. *World Neurosurg*. 2020;134:e636-e641. doi:10.1016/j.wneu.2019.10.160

48.	Sivakumar W, Guan J, Langevin JP, Barkhoudarian G, Kelly DF, Martin N. Enhanced recovery after brain tumor surgery: pilot protocol implementation in a large healthcare system. *Neurosurg Focus*. 2023;55(6):E5. doi:10.3171/2023.9.FOCUS23563

49.	Staartjes VE, Spinello A, Schwendinger N, Germans MR, Serra C, Regli L. Safety and Effectiveness of an Enhanced Recovery Protocol in Patients Undergoing Burr Hole Evacuation for Chronic Subdural Hematoma. *Neurosurgery*. 2024;95(1):146-157. doi:10.1227/neu.0000000000002849

50.	Sughrue ME, Bonney PA, Choi L, Teo C. Early Discharge After Surgery for Intra-Axial Brain Tumors. *World Neurosurg*. 2015;84(2):505-510. doi:10.1016/j.wneu.2015.04.019

51.	Laan MT, Roelofs S, Van Huet I, Adang EMM, Bartels RHMA. Selective Intensive Care Unit Admission After Adult Supratentorial Tumor Craniotomy: Complications, Length of Stay, and Costs. *Neurosurgery*. 2020;86(1):E54-E59. doi:10.1093/neuros/nyz388

52.	Todd MM, Warner DS, Sokoll MD, et al. A prospective, comparative trial of three anesthetics for elective supratentorial craniotomy. Propofol/fentanyl, isoflurane/nitrous oxide, and fentanyl/nitrous oxide. *Anesthesiology*. 1993;78(6):1005-1020. doi:10.1097/00000542-199306000-00002

53.	Uchida K, Yasunaga H, Miyata H, et al. Impact of remifentanil use on early postoperative outcomes following brain tumor resection or rectal cancer surgery. *J Anesth*. 2012;26(5):711-720. doi:10.1007/s00540-012-1397-3

54.	Vedicherla SV, Chew ZH, Kanavathy DT, et al. Chronic subdural hematoma enhanced recovery pathway: targeting excellent outcomes. *Neurosurg Focus*. 2025;59(4):E6. doi:10.3171/2025.7.FOCUS25531

55.	Venkatraghavan L, Bharadwaj S, Au K, Bernstein M, Manninen P. Same-day discharge after craniotomy for supratentorial tumour surgery: a retrospective observational single-centre study. *Can J Anaesth J Can Anesth*. 2016;63(11):1245-1257. doi:10.1007/s12630-016-0717-8

56.	Wagner A, Quiring A, Do A, Heim M, Schneider G, Meyer B. Postoperative Monitoring After Elective Intracranial Surgery in a Postanesthesia Care Unit is Safe, Efficient, and Cost-Effective. *Neurocrit Care*. Published online July 21, 2025. doi:10.1007/s12028-025-02323-z

57.	Wang Y, Liu B, Zhao T, et al. Safety and efficacy of a novel neurosurgical enhanced recovery after surgery protocol for elective craniotomy: a prospective randomized controlled trial. *J Neurosurg*. 2019;130(5):1680-1691. doi:10.3171/2018.1.JNS171552

58.	Wang Y, Xue YF, Zhao BF, et al. Real-World Implementation of Neurosurgical Enhanced Recovery After Surgery Protocol for Gliomas in Patients Undergoing Elective Craniotomy. *Front Oncol*. 2022;12:860257. doi:10.3389/fonc.2022.860257

59.	Wang L, Cai H, Wang Y, et al. Enhanced recovery after elective craniotomy: A randomized controlled trial. *J Clin Anesth*. 2022;76:110575. doi:10.1016/j.jclinane.2021.110575

60.	Wu J, Zhang W, Chen J, Fei H, Zhu H, Xie H. Application of and Clinical Research on Enhanced Recovery After Surgery in Perioperative Care of Patients With Supratentorial Tumors. *Front Oncol*. 2021;11:697699. doi:10.3389/fonc.2021.697699

61.	Yakar F, Bakirarar B, Elbir Ç, et al. Applications of enhanced recovery after surgery protocols for unruptured anterior circulation aneurysms in tertiary-level healthcare institutions: a national study. *Neurosurg Focus*. 2023;55(6):E12. doi:10.3171/2023.9.FOCUS23546

62.	Yang X, Ma J, Li K, et al. A comparison of effects of scalp nerve block and local anesthetic infiltration on inflammatory response, hemodynamic response, and postoperative pain in patients undergoing craniotomy for cerebral aneurysms: a randomized controlled trial. *BMC Anesthesiol*. 2019;19(1):91. doi:10.1186/s12871-019-0760-4

63.	Yang Y, Ou M, Zhou H, et al. Effect of Scalp Nerve Block with Ropivacaine on Postoperative Pain in Patients Undergoing Craniotomy: A Randomized, Double Blinded Study. *Sci Rep*. 2020;10(1):2529. doi:10.1038/s41598-020-59370-z

64.	Zaed I, Marchi F, Milani D, Cabrilo I, Cardia A. Role of Enhanced Recovery after Surgery (ERAS) Protocol in the Management of Elderly Patients with Glioblastoma. *J Clin Med*. 2023;12(18):6032. doi:10.3390/jcm12186032

65.	Zarrin D, Baisiwala S, Im J, et al. Cost savings associated with a nurse driven mobilization protocol for recovery after cranial tumor resection. *Acta Neurochir (Wien)*. 2025;167(1):237. doi:10.1007/s00701-025-06641-1
